# LncRNA ckATP1A1-AS1 inhibits influenza A virus replication by mediating innate immune responses and suppressing viral nuclear import

**DOI:** 10.1128/jvi.00259-26

**Published:** 2026-07-24

**Authors:** Menglu Fan, Zhiyuan Liu, Lulu Deng, Yiqing Zheng, Yiran Zeng, Yijia Zhang, Juan Su, Jun Xia, Jihui Ping

**Affiliations:** 1 MOE Joint International Research Laboratory of Animal Health and Food Safety, Engineering Center of Animal Immunity of Jiangsu Province, College of Veterinary Medicine, Nanjing Agricultural University 261674, Nanjing, China; 2 Key Laboratory for Prevention and Control of Herbivorous Animal Diseases of the Ministry of Agriculture and Rural Affairs & Xinjiang Animal Disease Research Key Laboratory, Xinjiang Academy of Animal Sciences Institute of Veterinary Medicine 643800, Urumchi, China; University of Freiburg, Freiburg, Germany

**Keywords:** long non-coding RNA, antiviral immunity, viral nucleoprotein, nuclear import

## Abstract

Influenza A viruses (IAVs) pose an ongoing threat to humans and other species because of their zoonotic potential. Accumulating evidence has demonstrated that certain long non-coding RNAs (lncRNAs) exhibit differential expression during viral infection and modulate diverse facets of viral pathogenesis. As key regulatory RNAs, lncRNAs participate in fundamental physiological processes and disease progression via a wide array of functional interactions with DNA, RNA, and proteins. Here, we identified ckATP1A1-AS1 as an antiviral host lncRNA that is induced by IAV infection. Functional analyses demonstrated that ckATP1A1-AS1 overexpression restricted infection by multiple IAV subtypes, whereas ckATP1A1-AS1 knockdown enhanced viral replication. Mechanistically, during IAV infection, the transcription factor JUN transcriptionally activates ckATP1A1-AS1, which further enhances the expression of interferon-β and key interferon-stimulated genes, thereby positively regulating type I interferon immune responses. Furthermore, ckATP1A1-AS1 interacts directly with the viral nucleoprotein, competitively disrupting its binding to importin α5, impairing its oligomerization, and blocking the nuclear import of viral ribonucleoprotein complexes. Consequently, ckATP1A1-AS1 suppresses viral ribonucleoprotein assembly and reduces viral polymerase activity. These findings establish ckATP1A1-AS1 as a key antiviral lncRNA that restricts IAV replication by coordinating innate immune signaling and directly targeting several steps in the viral replication cycle.

**IMPORTANCE:**

Accumulating evidence indicates that host long non-coding RNAs (lncRNAs) play important roles in regulating virus–host interactions during influenza A virus (IAV) infection. However, the functions and mechanisms of action of most IAV-associated lncRNAs remain unclear. This study identifies the novel chicken antisense lncRNA ckATP1A1-AS1 as a key antiviral factor with a unique dual mechanism: it is transcriptionally activated by transcription factor JUN and, in turn, upregulates the expression of interferon-β and key interferon-stimulated genes to positively regulate the type I interferon immune response. It directly interacts with viral nucleoprotein, competitively disrupting the binding of nucleoprotein to importin α5 and impairing nucleoprotein oligomerization, thereby suppressing viral ribonucleoprotein assembly and reducing viral polymerase activity.

## INTRODUCTION

Influenza A virus (IAV) is a major threat to global public health and the poultry industry because of its high mutation rates and frequent genetic reassortment (1, 2). Among the various subtypes, the H9N2 low-pathogenic avian influenza virus infects a broad range of hosts spanning avian and mammalian species (3, 4). This subtype frequently serves as a genetic donor, facilitating the emergence of novel variants with increased pathogenicity and zoonotic potential (5). The IAV genome comprises eight segments of negative-sense single-stranded RNA, organized into viral ribonucleoprotein (vRNP) complexes (6). Each vRNP consists of viral RNA, the nucleoprotein (NP), and the heterotrimeric polymerases PB1, PB2, and PA (7). Because IAV replication and transcription occur exclusively within the host cell nucleus, nuclear translocation of vRNPs is a prerequisite for a productive infection (7, 8). Although PB2 and NP utilize the classical nuclear import pathway via interaction with importin α/β1 heterodimers, the PB1–PA dimer relies on a non-classical pathway mediated by RanBP5. Consequently, disrupting the nuclear import of vRNP or the oligomerization of NP is a potent strategy, by which the host can restrict viral propagation (9–11).

To counteract viral invasion, hosts have evolved a complex and highly coordinated innate immune defense system that constitutes the first line of antiviral protection (12). Unlike mammals, chickens naturally lack RIG-I and IRF3; thus, the sensing of cytosolic viral RNA predominantly depends on the MDA5-LGP2 axis (13). This process activates MAVS, leading to the recruitment of the TBK1–IKK complex and the phosphorylation and nuclear translocation of IRF7 and NF-κB, thereby inducing interferon (IFN)-β and interferon-stimulated genes (ISGs) to establish an antiviral state (14, 15). In addition to IRF7 and NF-κB, the MAPK–JNK-mediated AP-1 transcriptional axis plays a critical cooperative role in avian innate immunity against IAV (16). Pattern recognition receptor engagement concomitantly activates c-Jun N-terminal kinase (JNK) signaling, promoting phosphorylation of c-Jun and ATF-2 and formation of the AP-1 complex, thereby enhancing antiviral gene transcription. AP-1 acts synergistically with IRF7 and NF-κB at the IFN-β promoter to amplify the transcriptional activation of IFN-β (17, 18). This coordinated regulation not only ensures robust IFN responses but also underscores AP-1 as an indispensable component of the avian innate immune network.

Long non-coding RNAs (lncRNAs) are transcripts exceeding 200 nucleotides in length that lack significant protein-coding potential (19, 20). Antisense lncRNAs, a distinct class of lncRNAs transcribed from the opposite strand of protein-coding or non-coding genes, often regulate their sense counterparts through complex transcriptional or post-translational networks (21). Although the roles of lncRNAs in cancer and autoimmunity have been reported previously (22), their involvement in viral pathogenesis, particularly in innate immune responses and direct interference with viral assembly or transport, is an emerging field. Recent studies have identified IAV-induced lncRNAs, including TSPOAP1-AS1, a virus-promoting lncRNA that facilitates viral replication by regulating IFN-β transcription (23). Other lncRNAs participate directly in the regulation of viral RNA stability and in viral assembly and release. For example, lnc-RPS6P3 interacts with NP and inhibits its oligomerization (24). Despite these advances in understanding, the functional significance and precise molecular mechanisms of action of many virus-responsive antisense lncRNAs remain unclear.

To address this gap, this study investigated the underlying mechanism of action of the antisense lncRNA ckATP1A1-AS1 in IAV replication. To elucidate the effects of ckATP1A1-AS1 on IAV replication, we overexpressed and knocked down the ckATP1A1-AS1 gene in DF-1 cells to analyze its interaction with other molecules and determine its regulatory effects on the signaling pathways in response to IAV infection. Our findings provide a more comprehensive understanding of the role of antisense lncRNAs in innate immunoregulatory pathways and the molecular mechanisms whereby antisense lncRNAs modulate IAV replication.

## RESULTS

### Infection with several types of viruses can induce ckATP1A1-AS1 expression

**Fig 1:**
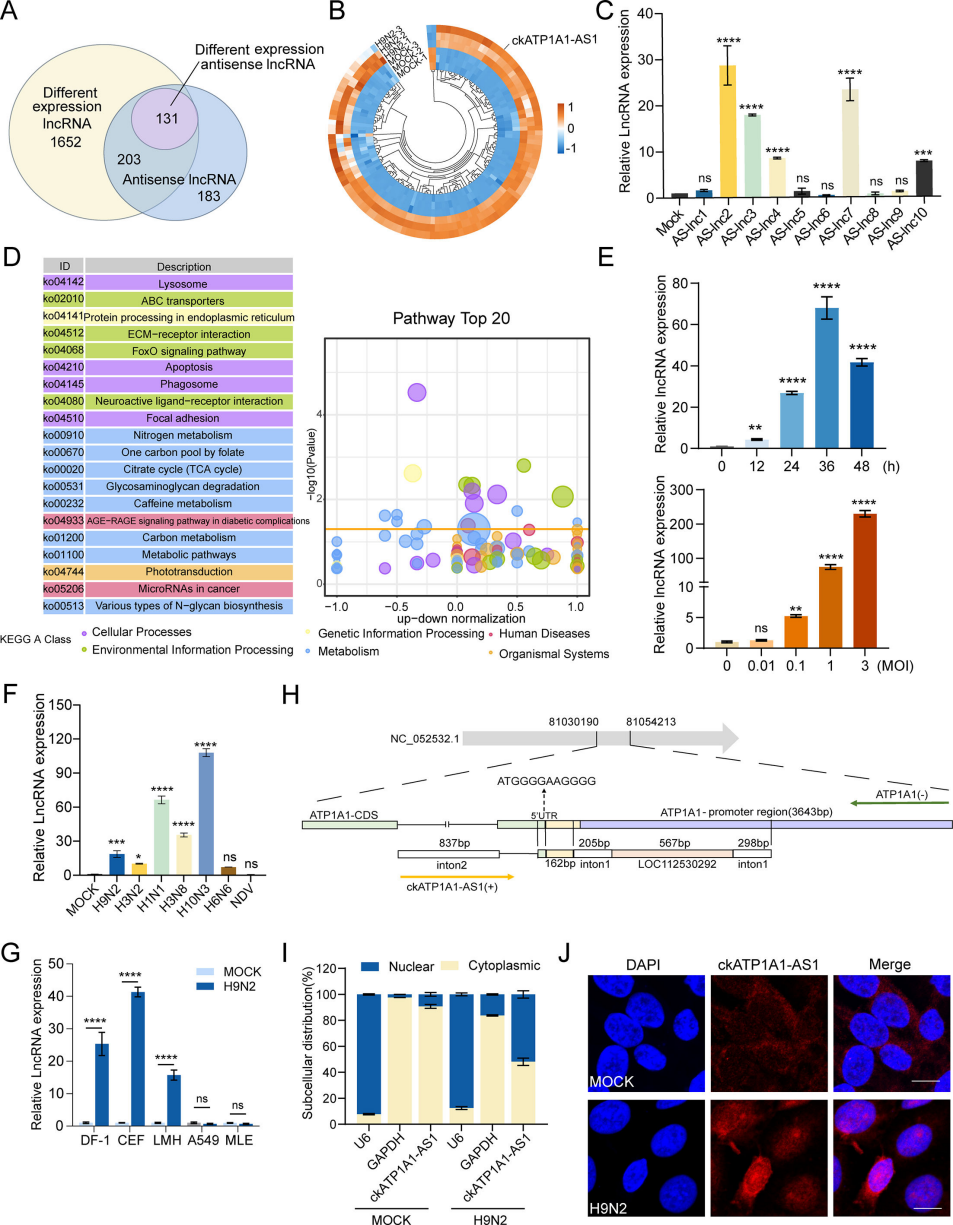
Identification and characterization of the virus-induced antisense lncRNA ckATP1A1-AS1 in DF-1 cells. (**A**) Venn diagram illustrating the quantitative distribution of differentially expressed lncRNAs, antisense lncRNAs, and differentially expressed antisense lncRNAs derived from whole-transcriptome sequencing data. (**B**) Circular heatmap of the expression profiles of differentially expressed antisense lncRNAs in mock- and H9N2-infected DF-1 cells. (**C**) RT-qPCR validation of selected antisense lncRNAs in DF-1 cells infected with H9N2 (MOI = 1) after 24 h. (**D**) Kyoto Encyclopedia of Genes and Genomes (KEGG) pathway enrichment analysis of DEGs whose expression levels were correlated with AS-lnc2 during H9N2 infection. (**E**) Results of RT-qPCR experiments to detect the expression levels of AS-lnc2 in DF-1 cells after infection with H9N2 virus at an MOI of 0.1 and sampling at different time points (0, 12, 24, 36, and 48 h) after infection (upper) or at different MOIs (0, 0.01, 0.1, 1, and 3) followed by 24 h of incubation (lower). (**F**) Induction of AS-lnc2 expression in DF-1 cells infected with different viruses, including distinct IAV subtypes and NDV. (**G**) Expression levels of ckATP1A1-AS1 detected by RT-qPCR at 24 h after different cell lines (DF-1, CEF, LMH, A549, and MLE cells) were infected with H9N2 virus (MOI = 1). (**H**) Genomic organization of ckATP1A1-AS1 at the ATP1A1 locus. (**I**) Subcellular localization of ckATP1A1-AS1 determined by nuclear-cytoplasmic fractionation followed by RT-qPCR analysis in mock- and IAV-infected DF-1 cells. GAPDH and U6 mRNA were used as cytoplasmic and nuclear controls, respectively. (**J**) RNA FISH analysis of the subcellular distribution of ckATP1A1-AS1 in DF-1 cells. The blue fluorescence represents DAPI-stained nuclei, whereas the red fluorescence represents ckATP1A1-AS1. All shown RT-qPCR results are normalized to GAPDH. Differences between groups were analyzed using one- or two-way ANOVA. Data are presented as the mean ± standard deviation (SD) of three independent experiments. *, *P* < 0.05; **, *P* < 0.01; ***, *P* < 0.001; ****, *P* < 0.0001; ns, no significant difference.

To systematically identify virus-responsive antisense lncRNAs, we first analyzed whole-transcriptome profiles of DF-1 cells infected with H9N2 IAV. DF-1 cells were infected at a multiplicity of infection (MOI) of 3, and total RNA was harvested at 12 h post-infection (hpi) for transcriptome sequencing. These experimental conditions were chosen to achieve efficient and relatively synchronized infection. In preliminary assays, NP immunofluorescence (IF) showed that most of the DF-1 cells were infected at 12 hpi, and viral RNA was readily detected, whereas severe cytopathic effects were not yet apparent. In total, 1,986 differentially expressed lncRNAs were identified, of which 517 were classified as antisense lncRNAs. Of these, 131 exhibited differential expression upon viral infection (Fig. 1A). Circular heatmap analysis revealed that most of these differentially expressed antisense lncRNAs were markedly upregulated following H9N2 virus infection (Fig. 1B). Based on their basal expression levels in DF-1 cells and the magnitude of induction upon viral infection, 10 antisense lncRNAs were selected for validation. Reverse-transcription quantitative real-time PCR (RT-qPCR) revealed that one candidate, designated AS-lnc2, was robustly induced by approximately 30-fold at 24 h following H9N2 infection (Fig. 1C). Furthermore, AS-lnc2 exhibited relatively high basal expression levels in DF-1 cells. To explore the potential biological relevance of AS-lnc2 during IAV infection, we performed a correlation-based trans-association analysis between AS-lnc2 and differentially expressed protein-coding genes. AS-lnc2-associated differentially expressed genes (DEGs) were primarily enriched in pathways related to lysosome function, ABC transporters, protein processing in the endoplasmic reticulum, ECM–receptor interactions, and FoxO signaling (Fig. 1D). Next, we characterized the induction kinetics of AS-lnc2 expression following viral infection. Time-course analysis revealed that AS-lnc2 expression was already upregulated at 12 hpi and continued to increase progressively as IAV infection proceeded. Infection of DF-1 cells with increasing MOIs of IAV resulted in the dose-dependent upregulation of AS-lnc2, with expression levels reaching up to approximately 200-fold induction at the highest MOI tested (Fig. 1E). In addition to the H9N2 subtype, other IAV subtypes, including H1N1, H3N2, H10N3, H3N8, and H6N6, also upregulated AS-lnc2 expression in DF-1 cells. Among these, H6N6 infection of DF-1 cells resulted in a relatively low and nonsignificant increase in AS-lnc2 expression. Furthermore, infection of DF-1 cells with Newcastle disease virus (NDV) resulted in a nonsignificant decrease in AS-lnc2 expression (Fig. 1F).

Next, we assessed the conservation of AS-lnc2 expression across different cell types. AS-lnc2 was detectable and inducible by H9N2 infection in avian-derived cells, including DF-1, CEF, and LMH cells, indicating conservation among avian species. In contrast, no AS-lnc2 expression was detected in mammalian cells, such as A549 and MLE, under the same conditions. These results suggest that AS-lnc2 exhibits conserved expression only among avian-derived cells (Fig. 1G). Genomic localization analysis revealed that AS-lnc2 is transcribed in the antisense head-to-head orientation relative to the ATP1A1 locus, with ATP1A1 serving as its sense counterpart. AS-lnc2 consists of two exons separated by an intron, and part of its sequence overlaps with the ATP1A1 promoter region in a reverse-complementary manner (Fig. 1H). Accordingly, AS-lnc2 was renamed chicken ATP1A1 antisense lncRNA 1 (ckATP1A1-AS1). Secondary structure prediction using RNAfold indicated that ckATP1A1-AS1 adopts a configuration, with folded terminal regions connected by a central stem (Fig. S1). Subcellular fractionation followed by RT-qPCR analysis revealed that ckATP1A1-AS1 was localized predominantly in the cytoplasmic compartment of DF-1 cells. Upon IAV infection, the total abundance of ckATP1A1-AS1 in the nuclear fraction increased by approximately 40% (Fig. 1I). The subcellular distribution of ckATP1A1-AS1 in DF-1 cells was further confirmed via RNA-fluorescence *in situ* hybridization (FISH) analysis (Fig. 1J).

Collectively, these results demonstrate that ckATP1A1-AS1 is a highly virus-responsive antisense lncRNA. Its expression is induced by infection with various IAVs, exhibits clear time- and dose-dependent induction patterns, and undergoes dynamic changes in subcellular distribution upon viral infection.

### ckATP1A1-AS1 inhibits IAV replication

**Fig 2:**
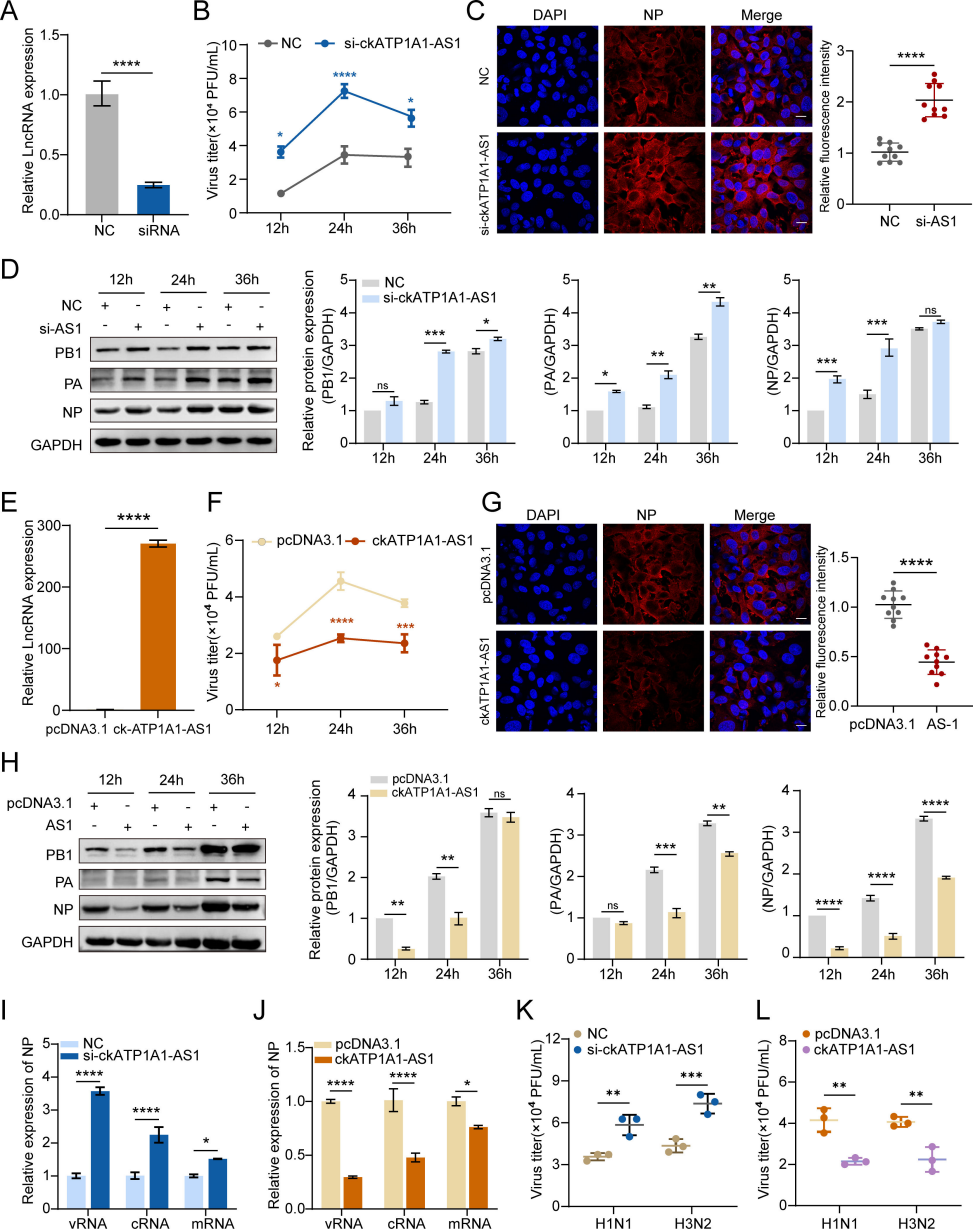
ckATP1A1-AS1 inhibits IAV replication in DF-1 cells. (**A**) Knockdown efficiency of ckATP1A1-AS1 in DF-1 cells transfected with specific siRNAs or the negative control (si-NC), as determined by RT-qPCR (left). (**B**) DF-1 cells with silenced ckATP1A1-AS1 were infected with H9N2 virus at an MOI of 0.1. Cell supernatants were collected at 12, 24, and 36 hpi, and viral titers were determined using the plaque assay. (**C**) An Indirect IF assay of viral protein NP expression in DF-1 cells following ckATP1A1-AS1 knockdown, with quantitative analysis of protein fluorescence intensity, was performed using ImageJ software (*n* = 10). (**D**) Western blotting analysis of IAV PB1, PA, and NP protein levels in DF-1 cells transfected with si-NC or si-ckATP1A1-AS1 at 12, 24, and 36 hpi. Protein levels were quantitatively analyzed, with GAPDH serving as the internal reference (*n* = 3). (**E**) The overexpression efficiency of ckATP1A1-AS1 in DF-1 cells transfected with pcDNA3.1-ckATP1A1-AS1 or the empty vector was determined by RT-qPCR (left) and RT-PCR (right). (**F**) DF-1 cells overexpressing ckATP1A1-AS1 were infected with H9N2 virus at an MOI of 0.1. Cell supernatants were collected at 12, 24, and 36 h post-infection, and viral titers were determined using the plaque assay. (**G**) IFA of viral protein NP expression in DF-1 cells following ckATP1A1-AS1 overexpression, with quantitative analysis of protein fluorescence intensity, was performed using ImageJ software (*n* = 10). (**H**) Western blot analysis of IAV PB1, PA, and NP protein levels in DF-1 cells transfected with pcDNA3.1-ckATP1A1-AS1 or the empty vector at 12, 24, and 36 hpi. (**I and J**) DF-1 cells with ckATP1A1-AS1 knockdown (**I**) or overexpression (**J**) were infected with H9N2 virus at an MOI of 1 for 24 h. The levels of cRNA, vRNA, and mRNA derived from IAV NP were quantitatively analyzed using RT-qPCR. (**K and L**) DF-1 cells with ckATP1A1-AS1 knockdown (**K**) or overexpression (**L**) were infected with H1N1 or H3N2 virus at an MOI of 0.1. Cell supernatants were collected at 24 h post-infection, and viral titers were determined using the plaque assay. All shown RT-qPCR results are normalized to GAPDH. Differences between groups were analyzed using one- or two-way ANOVA. Data are presented as the mean ± standard deviation (SD) of three independent experiments. *, *P* < 0.05; **, *P* < 0.01; ***, *P* < 0.001; ****, *P* < 0.0001; ns, no significant difference.

Given that ckATP1A1-AS1 was markedly upregulated in IAV-infected DF-1 cells, we next assessed whether modulation of ckATP1A1-AS1 expression affects IAV replication. After transfection of DF-1 cells with ckATP1A1-AS1-targeting small interfering RNA (siRNA), ckATP1A1-AS1 expression decreased by approximately 80% (Fig. 2A; Fig. S2A). Time-course analysis revealed that, relative to cells transfected with the negative control siRNA, ckATP1A1-AS1 knockdown resulted in higher viral titers at 12, 24, and 36 hpi (Fig. 2B; Fig. S2B). The regulatory effect of ckATP1A1-AS1 on IAV replication was confirmed via indirect IF analysis, demonstrating higher in viral protein fluorescence intensity upon ckATP1A1-AS1 knockdown (Fig. 2C). Western blotting revealed that knockdown of ckATP1A1-AS1 moderately enhanced the expression levels of the viral proteins PB1 and NP at 12, 24, and 36 hpi (Fig. 2D). Because ckATP1A1-AS1 was enriched in the nucleus during viral infection, we further used an antisense oligonucleotide (ASO)-based silencing approach to improve knockdown efficiency and validate the loss-of-function phenotype. ASO-mediated targeting of ckATP1A1-AS1 reduced its expression by approximately 85% at 24 hpi (Fig. S2C). Plaque assays further demonstrated that ckATP1A1-AS1 depletion enhanced H9N2 replication relative to the negative control, providing additional evidence that endogenous ckATP1A1-AS1 contributes to the restriction of IAV replication. Given that ckATP1A1-AS1 knockdown enhanced IAV replication, we also examined the effects of ckATP1A1-AS1 overexpression on viral replication. The pcDNA3.1-ckATP1A1-AS1 expression plasmid was constructed, and RT-PCR and RT-qPCR verified the ability of this plasmid to upregulate ckATP1A1-AS1 expression (Fig. 2E; Fig. S2E). Plaque assays demonstrated that ckATP1A1-AS1 overexpression resulted in lower IAV titers at 24 and 36 hpi (Fig. 2F; Fig. S2F). Consistent with this, indirect IF analysis revealed a reduction in NP protein fluorescence intensity upon ckATP1A1-AS1 overexpression (Fig. 2G). Western blotting analysis revealed that ectopic expression of ckATP1A1-AS1 partially suppressed the accumulation of viral PB1 and NP proteins at 12 hpi and 24 hpi (Fig. 2H). At the level of viral RNA synthesis, RT-qPCR analysis indicated that ckATP1A1-AS1 affected the abundance of NP-derived cRNA, vRNA, and mRNA at 24 hpi (Fig. 2I and J).

To exclude potential off-target effects of siRNAs and validate the antiviral function of ckATP1A1-AS1, we performed a rescue experiment. DF-1 cells were transfected with siRNA for ckATP1A1-AS1 knockdown, followed by co-transfection with the ckATP1A1-AS1 expression plasmid. Overexpression of ckATP1A1-AS1 effectively restored the siRNA-induced increase in viral replication, returning viral titers to levels equivalent to those in control, thus confirming the specific antiviral role of ckATP1A1-AS1 (Fig. S2G). Given that ckATP1A1-AS1 expression was induced by various IAV subtypes, we further examined whether ckATP1A1-AS1 exerts similar antiviral effects against other IAV strains. Plaque assays revealed that both the knockdown and overexpression of ckATP1A1-AS1 modulated the replication of H1N1 and H3N2 viruses, with trends consistent with those observed in H9N2 infection (Fig. 2K and L). Collectively, these results demonstrate that ckATP1A1-AS1 induction effectively restricts IAV replication across multiple viral subtypes.

### Antiviral activity of ckATP1A1-AS1 depends on the 838–1280nt domain

**Fig 3:**
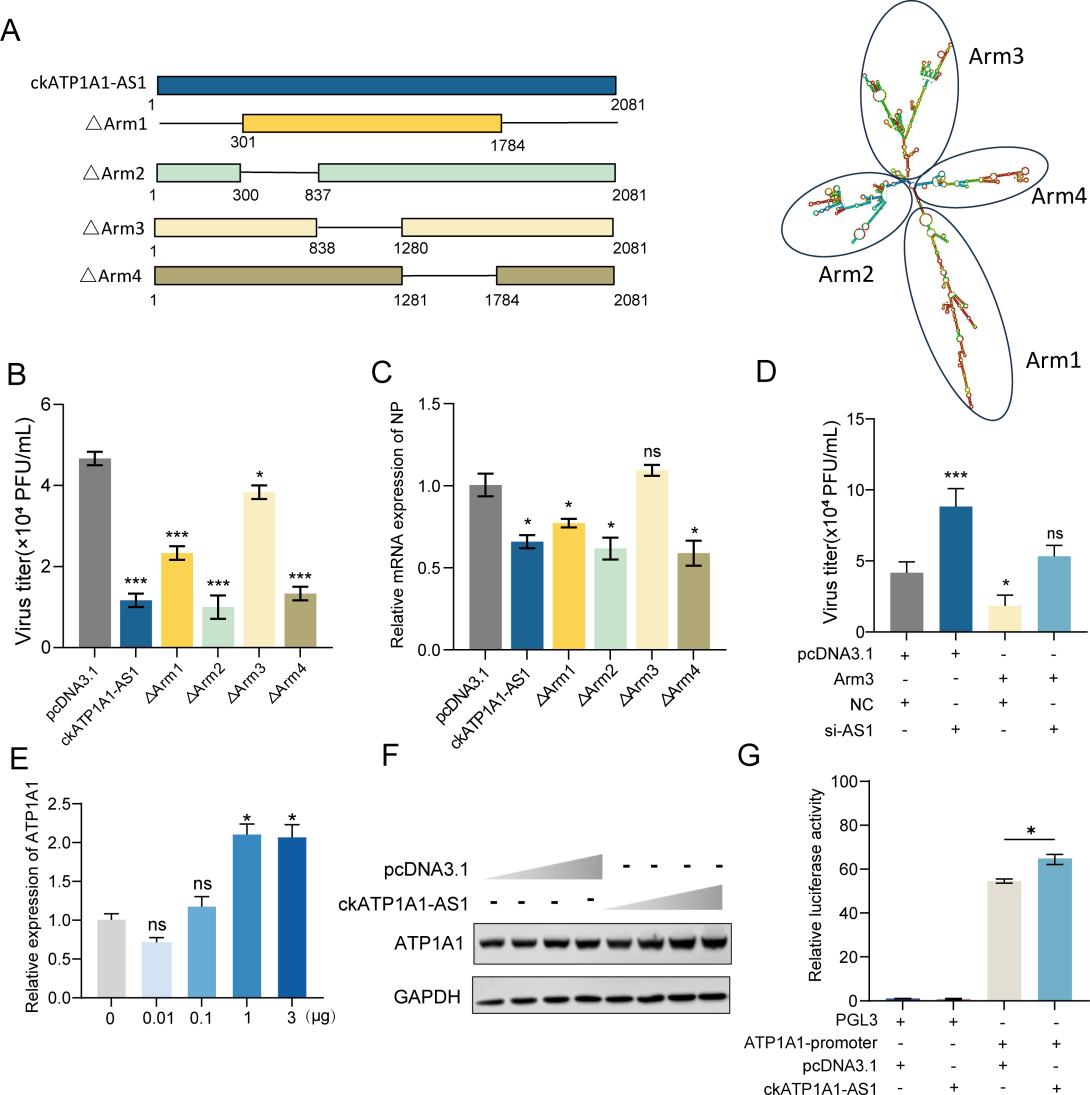
The Arm3 domain is essential for the antiviral activity of ckATP1A1-AS1, which functions independently of its host gene ATP1A1. (**A**) Schematic representation of the full-length ckATP1A1-AS1 and its four truncated mutants (ΔArm1 to ΔArm4), based on predicted secondary structures. (**B**) DF-1 cells overexpressing full-length ckATP1A1-AS1 or the indicated truncated mutants were infected with H9N2 virus at an MOI of 0.1 for 24 h, and viral titers were determined using the plaque assay. (**C**) RT-qPCR analysis of IAV NP mRNA levels in cells transfected with the specified ckATP1A1-AS1 constructs. (**D**) Following separate or concurrent ckATP1A1-AS1 knockdown (si-ckATP1A1-AS1) and Arm3 overexpression in DF-1 cells, the cells were infected with the H9N2 virus (MOI = 0.1) for 24 h. Subsequently, viral titers were determined using the plaque assay. (**E and F**) Expression kinetics of the host gene ATP1A1 during IAV infection. ATP1A1 mRNA levels were measured using RT-qPCR at the indicated time points (**E**), and ATP1A1 protein levels were analyzed by western blotting (**F**). (**G**) 293T cells were co-transfected with the pGL-ATP1A1-promoter-luc and pcDNA3.1-ckATP1A1-AS1 plasmids. Cell lysates were collected at 36 h after transfection, and the effects of ckATP1A1-AS1 on ATP1A1 promoter activity were determined using a dual-luciferase reporter assay. All shown RT-qPCR results are normalized to GAPDH. Differences between groups were analyzed using one-way ANOVA. Data are presented as the mean ± standard deviation (SD) of three independent experiments. *, *P* < 0.05; ***, *P* < 0.001; ns, no significant difference.

Antisense lncRNAs exert unique biological functions by acting as transcription factors (TFs) or utilizing specific hairpin structures to modulate the transcription of their host genes. To elucidate the mechanism by which ckATP1A1-AS1 inhibits IAV replication and to determine whether it regulates its host gene, we constructed four truncated mutants based on its sequence characteristics and predicted their secondary structures (Fig. 3A). Functional assays using DF-1 cells revealed that ectopic expression of full-length ckATP1A1-AS1 inhibited IAV replication, whereas the deletion of the Arm3 domain (838–1280nt, △Arm3 mutant) abolished this antiviral effect, with viral titers recovering to control levels (Fig. 3B). Consistent with this finding, RT-qPCR revealed that the ΔArm3 mutant failed to reduce the mRNA levels of the IAV NP gene (Fig. 3C). To further confirm the antiviral activity of Arm3, a rescue experiment was conducted by transfecting DF-1 cells with siRNA for ckATP1A1-AS1 knockdown, followed by co-transfection with a plasmid for Arm3-overexpression. In these experiments, pcDNA3.1-Arm3 overexpression effectively reversed the siRNA-mediated increase in viral replication, restoring viral titers to levels similar to those in the control group, thereby confirming that the truncated form of pcDNA3.1-Arm3 possesses antiviral activity (Fig. 3D). These findings demonstrate that the Arm3 domain is the primary functional region through which ckATP1A1-AS1 exerts its antiviral activity.

Sequence analysis indicated that the 850–1012nt region of ckATP1A1-AS1 (comprising Arm3 and Arm4) is antisense-complementary to the promoter and 5′ UTR regions of the host gene ATP1A1 (Fig. 1H). Thus, we monitored ATP1A1 expression kinetics during IAV infection. RT-qPCR analysis revealed that ATP1A1 mRNA levels remained stable during the early stages of infection (Fig. S3A). Western blotting confirmed that H9N2 infection increased ATP1A1 protein expression (Fig. S3B). Moreover, ATP1A1 overexpression restricted H9N2 replication at 24 and 36 hpi (Fig. S3C), suggesting that the host gene ATP1A1 also possesses intrinsic antiviral properties. Given that lncRNAs overlapping with host-gene regulatory regions can modulate host mRNA levels, we investigated whether ckATP1A1-AS1 and ATP1A1 act synergistically. Overexpression of ckATP1A1-AS1 led to a modest (approximately twofold) increase in ATP1A1 mRNA at a dose of 1 μg ckATP1A1-AS1, whereas further dose increases did not yield a corresponding increase in ATP1A1 expression (Fig. 3E). Consistent with this observation, ckATP1A1-AS1 overexpression did not alter ATP1A1 protein levels (Fig. 3F). Dual-luciferase reporter assays revealed that ckATP1A1-AS1 did not enhance ATP1A1 promoter activity (Fig. 3G). In summary, although both ckATP1A1-AS1 and its host gene ATP1A1 exhibit antiviral functions, ckATP1A1-AS1 relies on its 838–1280nt hairpin structure to restrict IAV replication, independent of its regulatory effect on the host gene ATP1A1.

### JUN as a critical transcription factor driving ckATP1A1-AS1 expression during IAV infection

**Fig 4:**
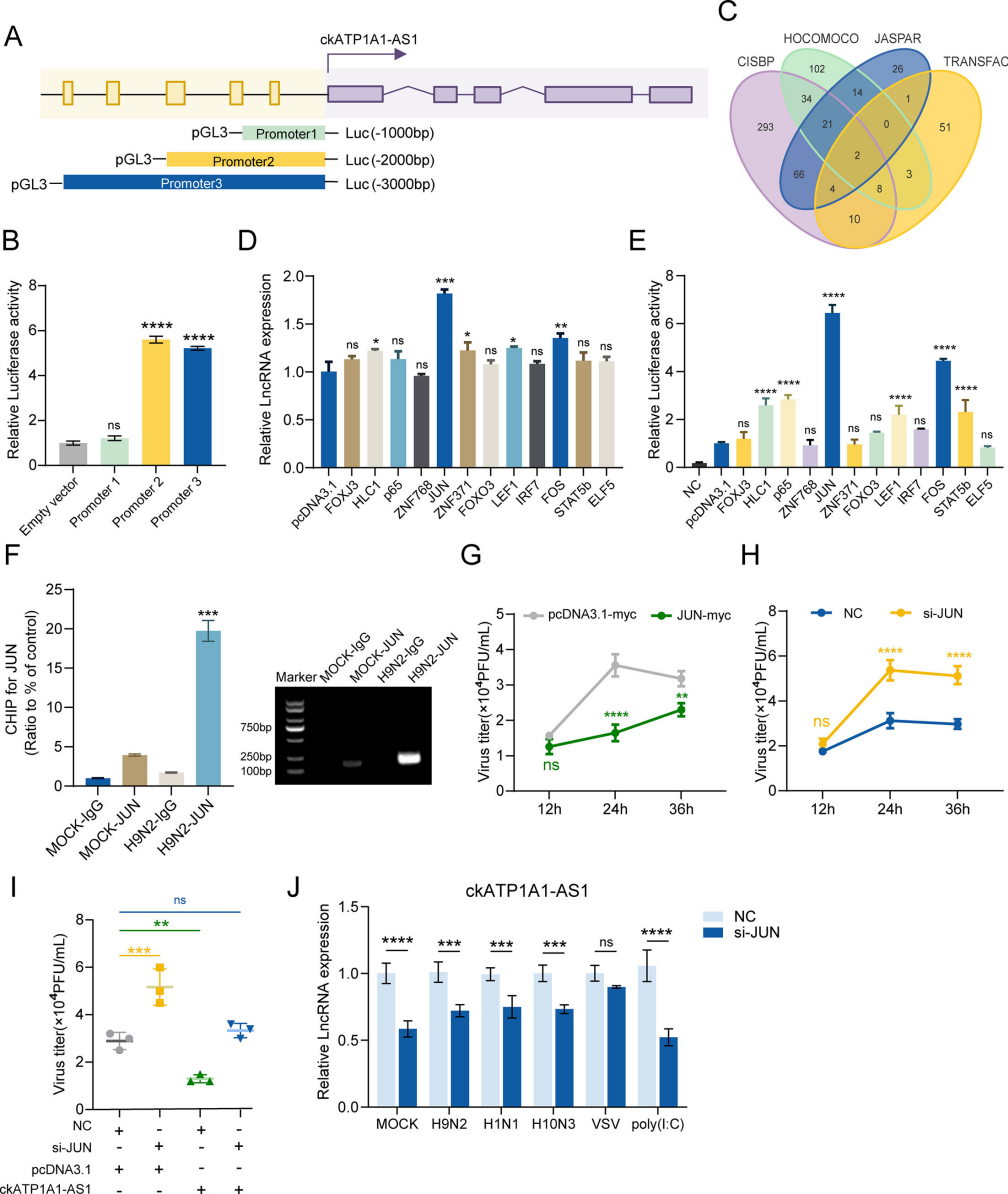
JUN acts as a key transcription factor driving ckATP1A1-AS1 expression. (**A**) Schematic representation of the ckATP1A1-AS1 genomic locus and the construction of three luciferase reporter plasmids containing truncated fragments of the ckATP1A1-AS1 promoter. (**B**) The promoter reporter plasmid of ckATP1A1-AS1 was transfected into 293T cells, and the core promoter region of ckATP1A1-AS1 in DF-1 cells was identified using the dual-luciferase reporter assay. (**C**) Venn diagram showing the overlap of TFs for the ckATP1A1-AS1 promoter predicted by several databases. (**D**) DF-1 cells were transfected with expression plasmids for 12 candidate TFs, and the endogenous mRNA levels of ckATP1A1-AS1 were determined by RT-qPCR. (**E**) Dual-luciferase reporter assay in 293T cells co-transfected with the ckATP1A1-AS1 promoter plasmid (pGL3-2000) and the indicated TF expression plasmids. (**F**) ChIP assay followed by qPCR and RT-PCR electrophoresis demonstrating the direct binding of JUN to the lncRNA promoter in the presence or absence of IAV infection. (**G and H**) Plaque assays showing the viral titers in the supernatants of JUN-overexpressing (**G**) and JUN-knockdown (**H**) cells at 12, 24, and 36 hpi. (**I**) Following separate or concurrent JUN knockdown (si-JUN) and ckATP1A1-AS1 overexpression in DF-1 cells, the cells were infected with the H9N2 virus (MOI = 0.1) for 24 h. Viral titers were determined using the plaque assay. (**J**) JUN knockdown (si-JUN) was performed in DF-1 cells, followed by infection with different viruses at an MOI of 1 for 24 h, or by stimulation with poly(I:C) for 12 h. Subsequently, total RNA was collected, and the expression levels of ckATP1A1-AS1 were determined by RT-qPCR. All shown RT-qPCR results are normalized to GAPDH. Differences between groups were analyzed using one- or two-way ANOVA. Data are presented as the mean ± standard deviation (SD) of three independent experiments. *, *P* < 0.05; **, *P* < 0.01; ***, *P* < 0.001; ****, *P* < 0.0001; ns, no significant difference.

The previous results indicated that ckATP1A1-AS1 may participate in the viral infection process via an ATP1A1-independent mechanism, but how ckATP1A1-AS1 functions during IAV infection and which upstream factors regulate its transcription remain unclear. To elucidate the transcriptional regulation of ckATP1A1-AS1, we first predicted its potential promoter region using bioinformatic tools and constructed three luciferase reporter plasmids containing truncated promoter fragments (pGL3-promoter-Luc) (Fig. 4A). Dual-luciferase reporter assays revealed that the 2,000 bp region upstream of the transcription start site exhibited the highest transcriptional activity, with sixfold greater luciferase activity than in the control group (Fig. 4B), thus establishing this 2,000 bp fragment as the core promoter region of ckATP1A1-AS1. Several databases were utilized to predict potential TFs binding to this core promoter region. Venn diagram analysis revealed that the candidate TFs were enriched in type I IFN-related signaling pathways (Fig. 4C). To identify the key regulators, 12 candidate TFs were selected for further validation. RT-qPCR revealed that JUN overexpression upregulated the expression of endogenous ckATP1A1-AS1 levels (Fig. 4D). Co-transfection experiments confirmed that JUN markedly enhanced ckATP1A1-AS1 promoter activity (Fig. 4E), indicating that JUN is the primary transcriptional activator of this lncRNA. To confirm the direct physical interaction between JUN and the ckATP1A1-AS1 promoter, we performed chromatin immunoprecipitation (ChIP) assays. JUN was found to bind specifically to the ckATP1A1-AS1 promoter, and this binding affinity was intensified upon IAV infection (Fig. 4F). Moreover, JUN mRNA levels increased in a time- and dose-dependent manner after IAV infection (Fig. S4A and B). Western blotting analysis further confirmed that the levels of phosphorylated JUN (p-JUN) were elevated at 12 and 24 hpi (Fig. S4C). These data indicate that IAV infection induces the expression and activation of JUN and promotes its recruitment to the ckATP1A1-AS1 promoter.

We also evaluated the functional contribution of JUN to the host antiviral response. Plaque assays revealed that JUN overexpression restricted IAV replication at several time points (Fig. 4G). In the plaque assay, the H9N2 IAV titer was higher in the JUN knockdown group than in the negative control (Fig. 4H). To determine whether the antiviral effect of ckATP1A1-AS1 was mediated by transcriptional activation of JUN, we performed a functional rescue assay. JUN knockdown increased the viral titer, whereas ckATP1A1-AS1 overexpression inhibited viral replication. Crucially, silencing JUN in ckATP1A1-AS1-overexpressing cells partially reversed the lncRNA-mediated viral inhibition, restoring the viral titer to a level equivalent to that of the control group (Fig. 4I). To determine whether JUN represents a shared upstream regulator of ckATP1A1-AS1 induction in response to viral stimuli, we examined ckATP1A1-AS1 expression in JUN-silenced DF-1 cells following infection with different IAV subtypes, VSV infection, or stimulation with poly(I:C) (a synthetic double-stranded RNA analog that mimics viral replication intermediates). JUN knockdown consistently attenuated ckATP1A1-AS1 induction by the tested IAV subtypes, indicating that JUN is necessary for IAV-triggered ckATP1A1-AS1 expression. Similarly, JUN depletion reduced poly(I:C)-induced ckATP1A1-AS1 expression, suggesting that stimulation by this viral RNA mimic activates ckATP1A1-AS1 at least partly through JUN. In contrast, ckATP1A1-AS1 expression after VSV infection was not substantially altered by JUN knockdown (Fig. 4J). These results indicate that JUN-dependent ckATP1A1-AS1 induction is preferentially associated with IAV infection and poly(I:C)-triggered viral RNA sensing. Collectively, these findings suggest that IAV infection triggers the expression and activation of JUN, which directly binds to the promoter to drive ckATP1A1-AS1 transcription.

### IAV-induced ckATP1A1-AS1 regulates type I IFN responses

**Fig 5:**
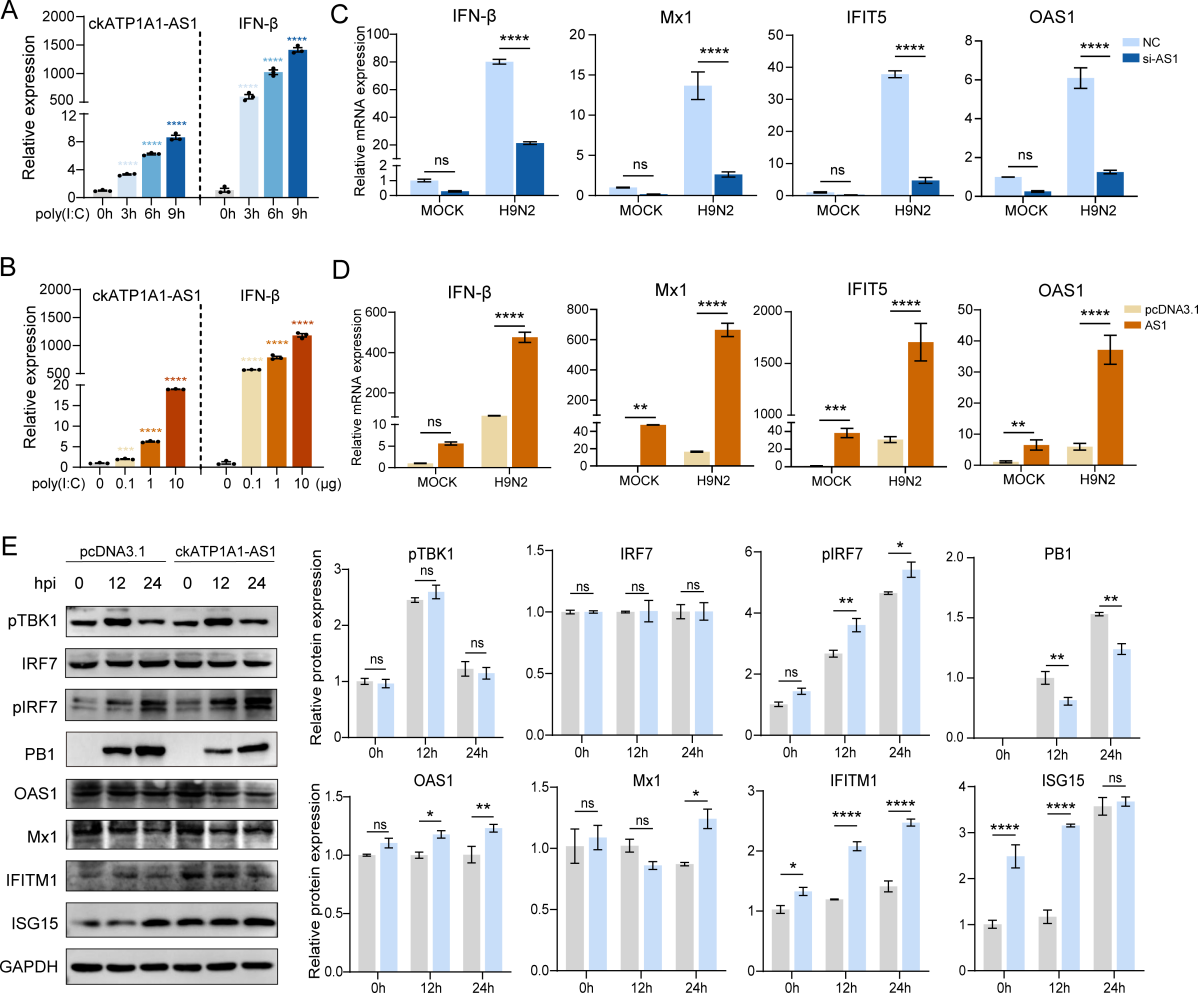
ckATP1A1-AS1 enhances the type I interferon response during viral infection. (**A and B**) DF-1 cells were transfected with poly(I:C) at the indicated time points (**A**) or at increasing concentrations (**B**). The mRNA levels of ckATP1A1-AS1 and IFN-β were determined by RT-qPCR. (**C**) Effects of ckATP1A1-AS1 knockdown on the transcription of IFN-β and ISGs, based on RT-qPCR. (**D**) Effects of ckATP1A1-AS1 overexpression on the transcription of IFN-β and ISGs, as analyzed using RT-qPCR. (**E**) Western blot analysis showing the effects of ckATP1A1-AS1 on signaling activation. DF-1 cells were transfected with pcDNA3.1-ckATP1A1-AS1 or empty vector, followed by H9N2 infection (MOI = 1). Protein levels were detected at the indicated time points and quantified, with GAPDH serving as the internal reference (*n* = 3). All RT-qPCR results are normalized to GAPDH. Differences between groups were analyzed using one- or two-way ANOVA. Data are presented as the mean ± standard deviation (SD) of three independent experiments. *, *P* < 0.05; **, *P* < 0.01; ***, *P* < 0.001; ****, *P* < 0.0001; ns, no significant difference.

Given that ckATP1A1-AS1 was induced within hours of infection with IAV, and that its transcription was mediated by JUN, we hypothesized that the antiviral activity of ckATP1A1-AS1 might be associated with the host’s innate immune response. To determine whether innate immune signaling directly drives ckATP1A1-AS1 upregulation, we transfected DF-1 cells with poly(I:C). Poly(I:C) induced ckATP1A1-AS1 expression in a time- and dose-dependent manner, exhibiting a kinetic profile highly synchronized with that of IFN-β in the positive control (Fig. 5A and B). These findings suggest that ckATP1A1-AS1 is a component of the innate immune-signaling network. To further characterize its role in the antiviral response, we assessed its regulatory effects on type I IFN production and on the expression of downstream effector genes. In both mock- and IAV-infected DF-1 cells, silencing of ckATP1A1-AS1 via siRNA reduced IFN-β secretion and downregulated the mRNA levels of type I IFN and various ISGs (Fig. 5C). Conversely, ckATP1A1-AS1 overexpression increased the transcriptional levels of these antiviral factors (Fig. 5D), thus identifying it as a positive regulator of the IFN response. We next investigated the effects of ckATP1A1-AS1 on IFN-related signaling pathways during viral infection. Western blotting analysis revealed that protein levels of the downstream antiviral effectors ISG15 and IFITM1 were elevated in the overexpression group at 12 and 24 hpi, with substantially reduced viral PB1 protein levels (Fig. 5E). Consistent with this result, in the negative control, IAV infection upregulated phosphorylated IRF7 (p-IRF7) in a time-dependent manner. The activation of p-IRF7 was further intensified in ckATP1A1-AS1 overexpressing cells, indicating that this lncRNA enhances antiviral signal transduction in host cells (Fig. 5E). However, TANK-binding kinase 1 (TBK1) activation was not altered by ckATP1A1-AS1 overexpression, indicating that ckATP1A1-AS1 does not act directly via the TBK1–IRF7 axis.

To further investigate whether ckATP1A1-AS1 exerts its antiviral effects through IRF7-mediated transcriptional activation of IFN-β, we performed a rescue experiment under conditions of IAV infection to delineate the regulatory relationship between ckATP1A1-AS1 and IRF7. In DF-1 cells, IRF7 was silenced via siRNA, ckATP1A1-AS1 was concurrently overexpressed, and IFN-β mRNA levels were measured. ckATP1A1-AS1 overexpression alone upregulated IFN-β expression, whereas IFN-β levels were reduced upon concurrent IRF7 knockdown, indicating that ckATP1A1-AS1-mediated transcriptional activation of IFN-β is at least partially dependent on IRF7 (Fig. S5A). Further assessment of viral titers in the culture supernatant revealed that IRF7 silencing enhanced IAV replication, whereas ckATP1A1-AS1 overexpression suppressed viral replication. Notably, in cells with concurrent ckATP1A1-AS1 overexpression and IRF7 knockdown, viral replication remained partially inhibited compared with the control group (Fig. S5B). These findings suggest that, in addition to functioning as a coactivator of IRF7-dependent IFN-β transcription, ckATP1A1-AS1 may also engage additional IRF7-independent antiviral pathways to restrict IAV replication.

Collectively, these findings confirm that ckATP1A1-AS1 amplifies transcriptional activation of IRF7 to boost the expression of type I IFNs and key ISGs, thereby positively regulating innate immunity and effectively suppressing the replication of various viruses, including IAV. However, the specific upstream regulatory pathways require further exploration.

### ckATP1A1-AS1 exerts an antiviral function in an early stage of IAV replication

**Fig 6:**
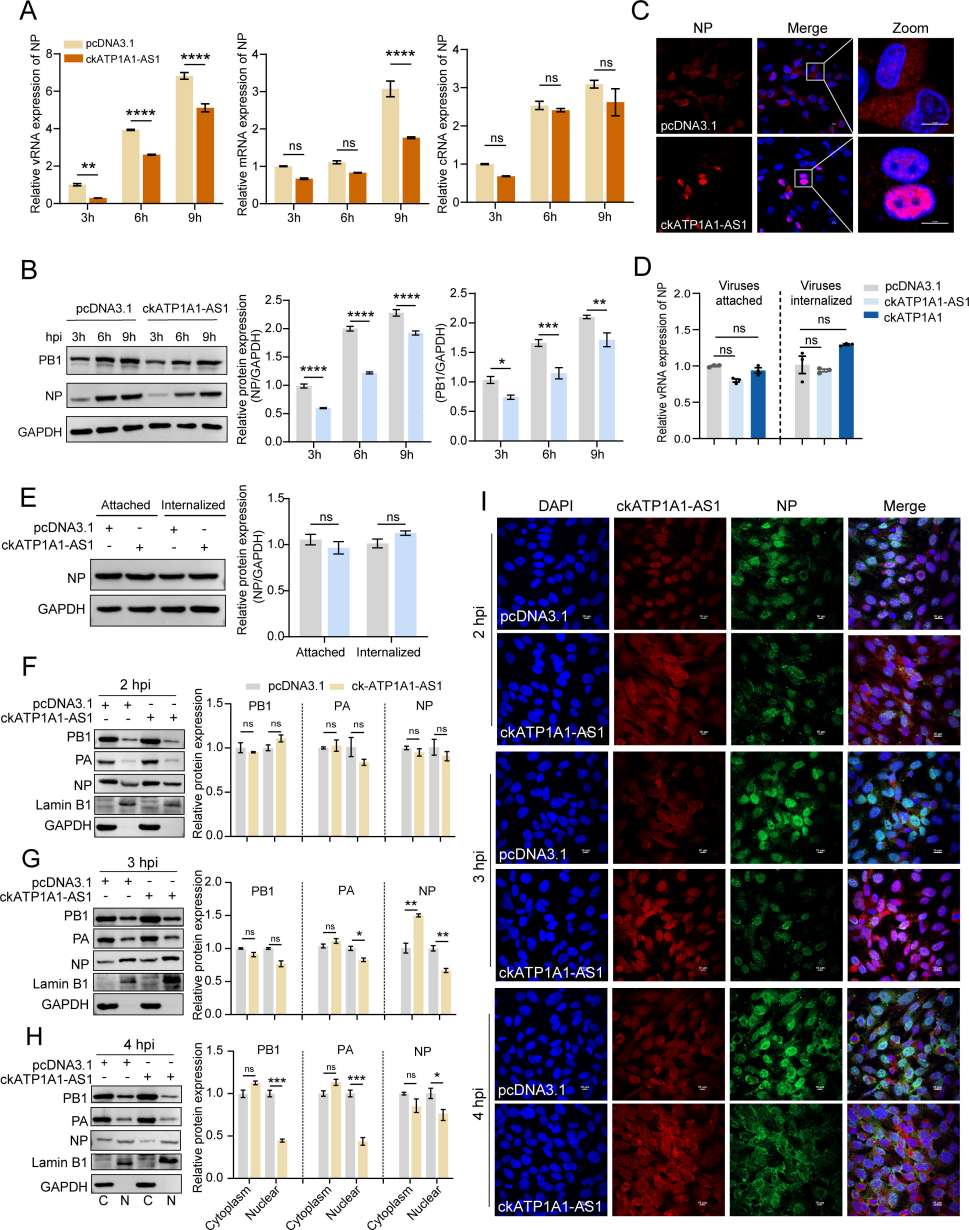
ckATP1A1-AS1 exerts an antiviral function in the early stage of viral replication. (**A**) RT-qPCR was used to detect the levels of NP gene vRNA, cRNA, and mRNA in DF-1 cells at 3, 6, and 9 hpi with H9N2 (MOI = 3). (**B**) Western blotting was used to detect the expression of NP and PB1 proteins at 3, 6, and 9 hpi with H9N2 (MOI = 3). Protein levels were quantitatively analyzed using ImageJ software, with GAPDH serving as the internal reference (*n* = 3). (**C**) IF assay was performed to observe the intracellular distribution of NP protein (red) at 3 hpi with H9N2 (MOI = 3). The cell nuclei were stained with DAPI (blue). (**D and E**) Evaluation of H9N2 virus attachment and internalization experiments. The NP vRNA level was detected by RT-qPCR (**D**), and the NP protein level was detected by western blot (**E**), respectively. The protein levels were quantitatively analyzed using ImageJ software, with GAPDH serving as the internal reference (*n* = 3). (F–H) Nuclear-cytoplasmic fractionation was detected in western blots at 2 (**F**), 3 (**G**), and 4 (**H**) hpi with H9N2 virus infection (MOI = 3). Lamin B1 and GAPDH were used as internal references for nuclear proteins and cytoplasmic proteins, respectively. The protein levels were quantitatively analyzed using ImageJ software. (**I**) IF assay to analyze the intracellular distribution of NP protein at 2, 3, and 4 hpi with H9N2 virus infection (MOI = 3). The cell nuclei are stained with DAPI (blue). All shown RT-qPCR results were normalized to GAPDH. Differences between groups were analyzed using one- or two-way ANOVA. Data are presented as the mean ± standard deviation (SD) of three independent experiments. *, *P* < 0.05; **, *P* < 0.01; ***, *P* < 0.001; ****, *P* < 0.0001; ns, no significant difference.

Various antiviral factors involved in innate immunity typically exert their functions against viral replication during the early phase of viral infection. Based on the aforementioned results, ckATP1A1-AS1 was upregulated in the early stage of viral infection. To investigate the specific stage at which ckATP1A1-AS1 exerts its antiviral effects, we first analyzed the kinetics of viral RNA synthesis. In DF-1 cells infected with H9N2 at an MOI of 3, ckATP1A1-AS1 overexpression reduced the levels of NP vRNA and cRNA as early as 3 hpi. At 9 hpi, the transcription of vRNA, cRNA, and mRNA was suppressed (Fig. 6A). Consistent with our transcriptomic analysis results, western blotting analysis revealed that ckATP1A1-AS1 downregulated the expression of viral NP and PB1 proteins starting at the early stage of infection (3 hpi) (Fig. 6B). IF assays at 3 hpi revealed that the nuclear entry of NP protein was markedly hindered in the ckATP1A1-AS1-overexpressing group (Fig. 6C). Next, we investigated whether this inhibitory effect occurred during the initial stages of infection, specifically during viral attachment or internalization. Based on RT-qPCR and western blotting analyses, neither NP vRNA levels nor NP or PB1 protein content varied between the ckATP1A1-AS1-overexpressing and control groups during these early entry steps (Fig. 6D and E). This indicates that ckATP1A1-AS1 does not interfere with viral attachment or internalization but acts at the post-entry stage. Given the reduction in viral proteins in the nucleus, we focused on the nuclear translocation of vRNP complexes. Via cell fractionation, we monitored the subcellular distribution of viral proteins during the early phase of infection (2–4 hpi). Although no significant differences were observed at 2 hpi, ckATP1A1-AS1 blocked the nuclear import of PB1, PA, and NP proteins at 3–4 hpi. Specifically, NP protein exhibited massive accumulation in the cytoplasm, and nuclear PA levels were substantially reduced in ckATP1A1-AS1-overexpressing cells (Fig. 6F through H). Likewise, indirect IF assays confirmed that overexpression of ckATP1A1-AS1 suppressed the nuclear import of IAV proteins. At 3 hpi with IAV, the viral NP protein exhibited marked colocalization with the nucleus in the control group. Conversely, in the ckATP1A1-AS1-overexpressing group, viral NP protein expression was reduced, and the fluorescent signal was predominantly distributed in the cytoplasm and perinuclear region. This distinct redistribution of the fluorescent signal was even more pronounced at 4 hpi. (Fig. 6I). Taken together, these results indicate that ckATP1A1-AS1 inhibits IAV infection not by blocking viral entry but by suppressing the early nuclear import of IAV, thereby repressing the initiation of viral genome replication and transcription.

### ckATP1A1-AS1 interacts with NP and impairs its association with importin α5

**Fig 7:**
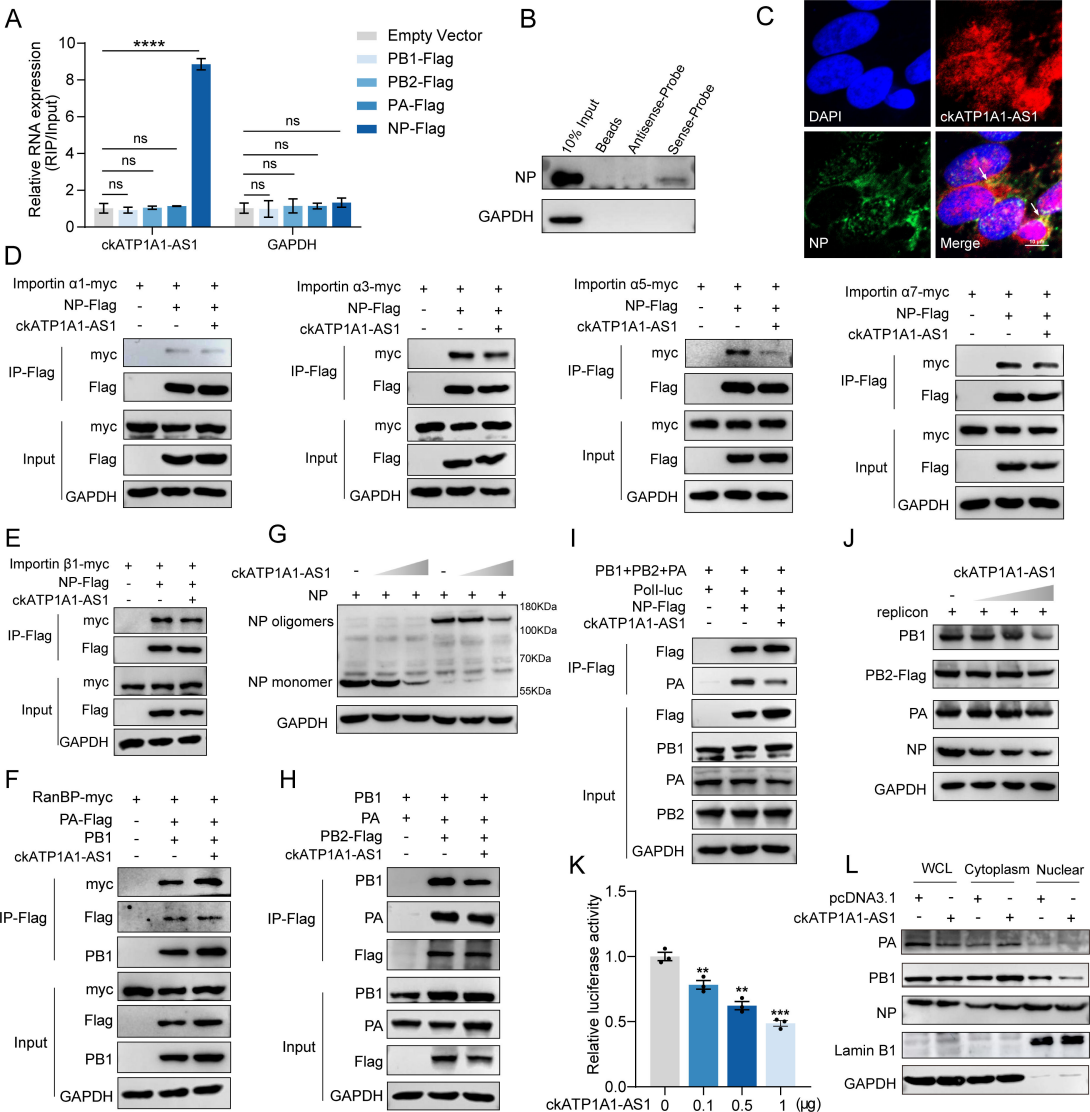
ckATP1A1-AS1 blocks the binding of NP to importin α5 and inhibits the polymerase activity of vRNP. (**A**) RIP assay in 293T cells co-transfected with ckATP1A1-AS1 and FLAG-tagged viral proteins (NP, PB1, PB2, or PA). (**B**) RNA pull-down assay using a specific ckATP1A1-AS1 probe, followed by western blotting for NP in H9N2-infected DF-1 cells. (**C**) Perinuclear co-localization of ckATP1A1-AS1 (red) and NP protein (green) at 6 hpi, visualized by RNA FISH combined with IF. (**D**) Co-IP assays examining the effects of ckATP1A1-AS1 on the interaction between FLAG-tagged NP and MYC-tagged importin α1, α3, α5, or α7. (**E**) Co-IP assays examining the effects of ckATP1A1-AS1 on the interaction between FLAG-tagged NP and MYC-tagged importin β1. (**F**) Co-IP assay assessing the interaction between FLAG-tagged PA and MYC-tagged RanBP5 in the presence of PB1 and ckATP1A1-AS1. (**G**) Western blot analysis to quantify NP homo-oligomerization. NP monomers and trimers were detected in cells with increasing expression levels of ckATP1A1-AS1. (**H**) Co-IP assay assessing the formation of the heterotrimeric polymerase complex PB1–PB2–PA in the presence of ckATP1A1-AS1. (**I**) Co-IP assay evaluating functional vRNP assembly. The interaction between NP and PA was analyzed in cells expressing ckATP1A1-AS1. (**J and K**) Viral polymerase activity determined by minigenome assay. Reporter protein levels (**J**) and luciferase activity (**K**) were measured following dose-dependent expression of ckATP1A1-AS1. (**L**) Subcellular distribution of NP, PB1, and PA in the minigenome system, analyzed using nucleocytoplasmic fractionation. GAPDH and Lamin B1 served as cytoplasmic and nuclear markers, respectively. Differences between groups were analyzed using one-way ANOVA. Data are presented as the mean ± standard deviation (SD) of three independent experiments. **, *P* < 0.01; ***, *P* < 0.001; ****, *P* < 0.0001; ns, no significant difference.

Many lncRNAs perform their biological functions by interacting with specific proteins. Given that ckATP1A1-AS1 restricts the nuclear import of vRNPs, we investigated whether it binds directly to viral RNP components. RNA immunoprecipitation (RIP) assays in 293T cells co-transfected with ckATP1A1-AS1 and FLAG-tagged viral proteins (NP, PB1, PB2, or PA) revealed that ckATP1A1-AS1 interacted specifically with NP but not with PB1, PB2, or PA (Fig. 7A). This interaction was further confirmed via RNA-protein pull-down assays, which detected an association between endogenous ckATP1A1-AS1 and NP (Fig. 7B). Combined RNA FISH and IF staining revealed distinct perinuclear co-localization of ckATP1A1-AS1 and NP at 6 hpi (Fig. 7C), suggesting that their interaction occurs near the nuclear envelope during the early stages of IAV infection. Nuclear import of NP is mediated by its binding to various nuclear import adapters, specifically importin α isoforms (α1, α3, α5, or α7) and subsequently to importin β1. To investigate whether ckATP1A1-AS1 acts as a competitive inhibitor, we performed co-immunoprecipitation (Co-IP) assays to evaluate the interactions between NP and these transporters. Although the binding of NP to importin α1, α3, α7, or β1 remained unaffected by ckATP1A1-AS1, its expression attenuated the interaction between NP and importin α5 (Fig. 7D and E). Although ckATP1A1-AS1 also suppresses the nuclear entry of PB1 and PA, these proteins do not directly interact with the lncRNA. As the PA–PB1 heterodimer utilizes a non-classical nuclear transport pathway mediated by RanBP5, we examined whether ckATP1A1-AS1 targets this mechanism. Co-IP assays revealed that ckATP1A1-AS1 overexpression did not alter the binding affinity between PB1 and RanBP5 (Fig. 7F). This result suggests that inhibition of PA–PB1 nuclear entry by ckATP1A1-AS1 is likely a secondary effect of blocking the nuclear import of the entire vRNP complex, rather than resulting from its direct interference with the RanBP5 transport machinery. These findings indicate that ckATP1A1-AS1 interferes specifically with the formation of the NP–importin α5 complex, thereby hindering the nuclear translocation of vRNP complexes.

### ckATP1A1-AS1 impairs vRNP complex assembly and suppresses IAV polymerase activity

IAV NP forms homo-oligomers that encapsulate the genomic RNA, thus providing the structural framework necessary for vRNP complex assembly. Therefore, we investigated whether ckATP1A1-AS1 affects the homo-oligomers of NP. Increasing ckATP1A1-AS1 expression led to a dose-dependent reduction in the levels of both NP monomers and trimers (Fig. 7G), suggesting that this lncRNA effectively inhibits NP homo-oligomerization. Although the RIP assays revealed no direct interaction between ckATP1A1-AS1 and polymerase proteins, we examined whether this lncRNA interferes with the formation of the PB1–PB2–PA heterotrimer. Co-IP assays using HEK293T cells revealed that the amounts of PB1 and PA that co-precipitated with FLAG-PB2 remained unchanged in the presence of ckATP1A1-AS1 (Fig. 7H). Because viral transcription and replication are catalyzed by the vRNP complex, we focused on whether the interaction between ckATP1A1-AS1 and NP impairs overall vRNP assembly. We reconstituted the vRNP complex in HEK293T cells and performed Co-IP assays. As PA does not interact directly with NP but is co-precipitated via the assembled vRNP complex, it serves as a marker for complex integrity. We found that the amount of PA co-precipitated with NP-FLAG was reduced upon ckATP1A1-AS1 overexpression (Fig. 7I), demonstrating that this lncRNA disrupts the assembly of functional vRNP complexes. Finally, we utilized a minigenome reporter assay to evaluate the functional effects of ckATP1A1-AS1 on viral polymerase activity. Viral polymerase activity declined dose-dependently with increasing ckATP1A1-AS1 expression (Fig. 7J). Consistent with this finding, luciferase reporter protein levels were downregulated by ckATP1A1-AS1 (Fig. 7K). Nucleocytoplasmic fractionation within the minigenome system confirmed that ckATP1A1-AS1 reduced the nuclear abundance of the PB1 and PA proteins (Fig. 7L). In summary, these findings demonstrate that ckATP1A1-AS1 effectively restricts IAV infection by impairing NP oligomerization and functional vRNP complex assembly, thereby suppressing viral polymerase activity at its source.

## DISCUSSION

**Fig 8:**
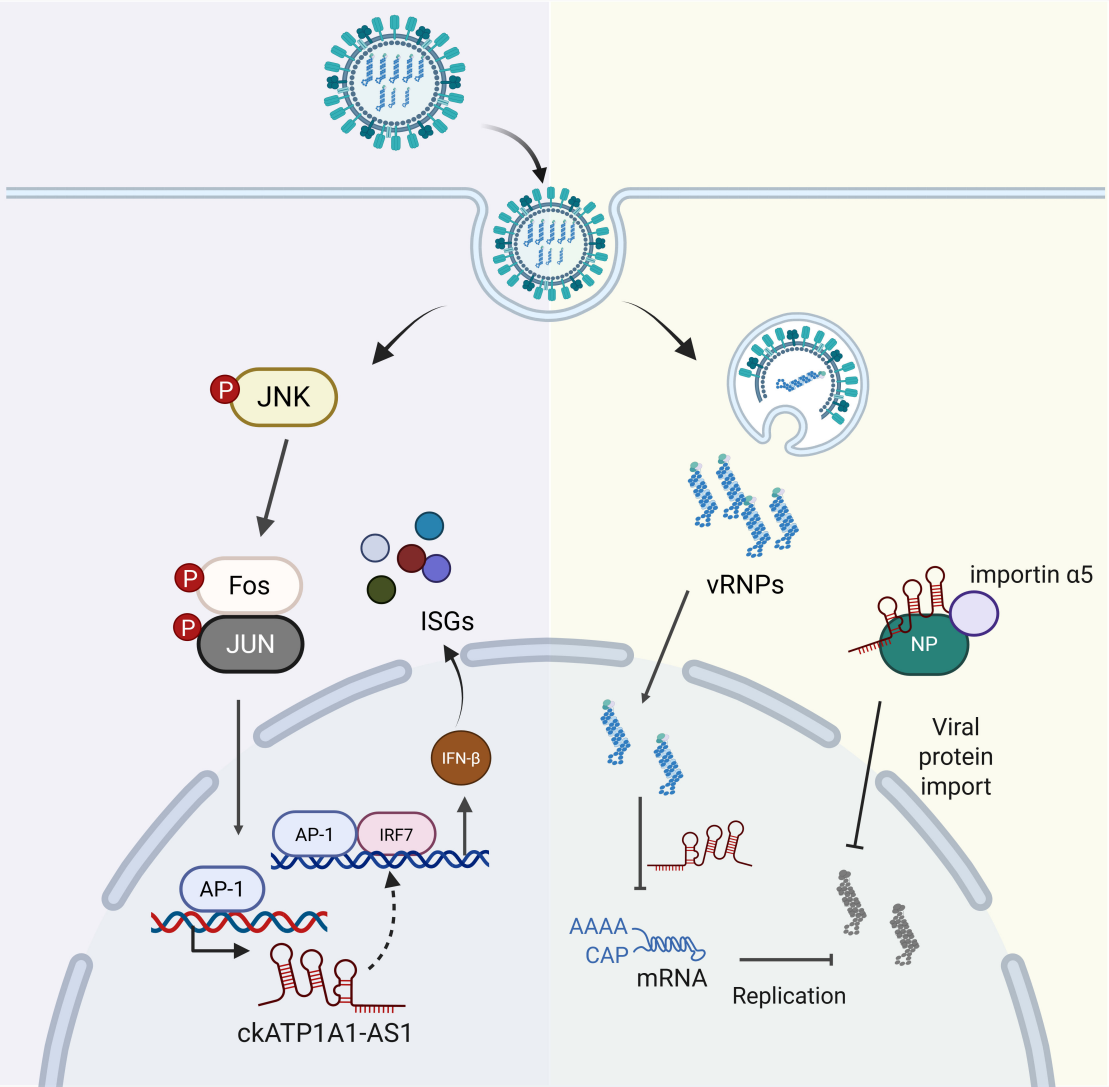
Working model in which ckATP1A1-AS1 inhibits IAV replication. Following infection with influenza A viruses, ckATP1A1-AS1 transcription is activated by the transcription factor JUN. As a transcriptional coactivator of IRF7, ckATP1A1-AS1 enhances the production of type I interferon and ISGs, thereby potentiating the innate immune response. Additionally, ckATP1A1-AS1 binds directly to the viral nucleoprotein, competitively inhibiting its interaction with importin α5. This disrupts the nuclear import of the viral ribonucleoprotein complex, impairs nucleoprotein oligomerization, and suppresses viral polymerase activity, ultimately restricting the replication of influenza A viruses.

Although the H9N2 subtype of avian influenza virus is categorized as a low-pathogenic influenza virus, it plays a pivotal role in the adaptive evolution and cross-species transmission of influenza viruses (25, 26). Recent advances in high-throughput transcriptome sequencing have revealed the differential expression of lncRNAs under various pathological conditions, including viral infections (27, 28). However, the functional roles of lncRNAs in avian hosts, particularly in chickens, remain poorly understood within the context of host–virus interaction networks. Here, we identified a novel antiviral host factor, ckATP1A1-AS1, that is upregulated upon viral infection. Functional assays demonstrated that ectopic expression of ckATP1A1-AS1 restricted the replication of various IAV subtypes, whereas its knockdown facilitated viral infection (Fig. 8). Unlike classical antiviral effectors that completely block viral replication, many host lncRNAs function as regulatory modulators that fine-tune the balance between viral propagation and innate immune responses (29, 30). Although the antiviral effect of ckATP1A1-AS1 was moderate rather than complete, we believe that this phenotype remains biologically meaningful in the context of host lncRNA-mediated antiviral regulation. ckATP1A1-AS1 reproducibly attenuated IAV replication across various experimental conditions and independent loss-of-function experiments. These findings identify ckATP1A1-AS1 as a critical endogenous molecule required by the host to restrict IAV infection.

ATP1A1-AS1 is also present in mammalian cells and has been implicated in the development and progression of cancer (31, 32). This raises an important question: whether ckATP1A1-AS1 and mammalian ATP1A1-AS1 represent evolutionarily conserved homologous lncRNA transcripts and whether ATP1A1-AS1 similarly possesses the ability to regulate IAV infection. Our comparative analysis showed that ckATP1A1-AS1 and human ATP1A1-AS1 are both transcribed from the antisense strand of the orthologous ATP1A1 locus, indicating positional relatedness. However, they differ markedly in genomic organization: ckATP1A1-AS1 is arranged in a convergent orientation and partially overlaps the chicken ATP1A1 promoter region, whereas human ATP1A1-AS1 displays a divergent head-to-head orientation (31). Consistent with this difference, pairwise sequence comparison revealed limited primary sequence conservation between ckATP1A1-AS1 and human ATP1A1-AS1. RNAalifold-based consensus folding analysis further suggested only putative local secondary-structure similarity rather than extensive full-length structural conservation (33). The predicted consensus structure exhibited high ensemble diversity and a very low frequency of the minimum free-energy structure, indicating that the full-length structural model should be interpreted cautiously and that any conserved structural features are more likely to be local rather than global (Fig. S6A and B). Functionally, human ATP1A1-AS1 failed to restrict IAV replication under the same experimental conditions (Fig. S6C). Thus, ckATP1A1-AS1 is best interpreted as an avian ATP1A1-associated antisense lncRNA with species- or lineage-specific antiviral activity, rather than a sequence-, structure-, or function-conserved ortholog of human ATP1A1-AS1.

In avian hosts, the IFN-I signaling pathway serves as the core innate immune mechanism for curbing IAV replication (34, 35). Members of the MAPK family, particularly the JNK pathway, are essential regulators of the host responses to influenza infection. Accumulation of viral RNA induces sustained JNK activation, leading to c-Jun phosphorylation and the formation of the AP-1 transcription complex (18, 36). Replication of IAV stimulates the transcriptional activity of the nuclear AP-1 factor and its target genes via the MKK4/7–JNK pathway, in which the NS1 protein can suppress JNK-dependent responses, thereby facilitating the efficient replication and spread of the virus (18). The present study identified ckATP1A1-AS1 as an AP-1-dependent antiviral lncRNA that exerts its anti-IAV activity by participating in the JNK–AP-1–type I IFN regulatory axis. Mechanistically, ckATP1A1-AS1 facilitates IRF7-mediated transcriptional activation of IFN-β and enhances the expression of key ISGs, including Mx1 and IFIT5. Notably, at the protein level, only ISG15 and IFITM1 revealed clear changes, whereas the differences in OAS1 and Mx1 expression were less pronounced. This discrepancy between mRNA and protein levels may be attributable to the limited recognition efficiency or specificity of commercially available antibodies against chicken proteins, and may also reflect differences in post-transcriptional regulation, protein stability, or the detection-time window of ISG expression. Nevertheless, the mRNA data support a positive regulatory role of ckATP1A1-AS1 in the induction of IFN-β and various ISGs, suggesting that ckATP1A1-AS1 contributes to enhancing the host’s innate antiviral response during IAV infection. Our promoter analysis further suggests that the JUN-responsive regulation of ckATP1A1-AS1 may not be broadly conserved across mammals. Although putative AP-1 and JUN-binding motifs were identified in the ckATP1A1-AS1 promoter and were supported by our JUN gain- and loss-of-function data, the corresponding promoter architecture is not clearly conserved in human ATP1A1-AS1. Together with the lack of antiviral activity of human ATP1A1-AS1, these findings suggest that JUN-driven induction of ckATP1A1-AS1 is likely an avian-adapted regulatory feature.

Beyond its role in nuclear IFN signaling, AP-1 plays a critical role in programmed cell death (37). Sustained AP-1 activation can transcriptionally activate pro-apoptotic genes, such as FasL, triggering the death receptor pathway (38, 39). KEGG enrichment analysis revealed that genes co-expressed with ckATP1A1-AS1 were enriched in pathways related to lysosomes, FoxO signaling, and apoptosis (Fig. S7). Based on validation of apoptosis-related genes with strong correlations, ckATP1A1-AS1 expression was positively correlated with FASLG levels but negatively correlated with TNFSF10 levels following H9N2 infection (Fig. S8A and B). Both the FASLG and TNFSF10 genes mediate the extrinsic apoptotic pathway (death receptor pathway), which relies primarily on the binding of death receptors to their ligands to activate caspases and plays a crucial role in host immune responses against viral infection (40, 41). Therefore, this lncRNA may also be involved in the cellular apoptotic pathway. Nonetheless, the precise molecular pathways through which ckATP1A1-AS1 modulates apoptosis and the potential involvement of other effector factors warrant further investigation.

The vRNP complex is central to the IAV replication cycle, with NP being the most abundant component and the primary carrier of viral RNA (42, 43). To achieve efficient replication, IAV exploits interactions between NP and various host factors (44–46). LncRNAs regulate diverse biological processes, including nuclear transport. For instance, the lncRNA IPAN binds to and stabilizes PB1, thereby promoting efficient RNA synthesis (47). Here, ckATP1A1-AS1 expression was elevated in the nucleus and perinuclear region following infection, suggesting its role in viral genome transport. Conversely, ckATP1A1-AS1 weakened the NP–importin α5 interaction, suggesting that this lncRNA may competitively bind to key domains of NP (such as the tail loop or the RNA-binding groove) to block monomer-to-oligomer conversion (48). Although these findings confirm its direct interaction with NP, the possibility that ckATP1A1-AS1 recruits other host proteins, such as KRT6A (49) or eEF1D (10), to coordinately regulate NP function remains an open question.

An MOI of 3 was used for transcriptomic profiling to achieve a robust and relatively synchronized infection, thereby facilitating the identification of IAV-associated transcriptional changes. Although this relatively high viral inoculum may not fully reflect natural infection conditions, subsequent functional analyses conducted using lower viral doses yielded consistent results. These findings support the robustness of our conclusions across different viral doses. In addition, although our experiments were conducted primarily in cultured avian cells, the induction of ckATP1A1-AS1 in multiple avian-derived cell types suggests that this lncRNA may participate more broadly in the host response to avian influenza virus infection. Mechanistically, ckATP1A1-AS1 integrates the amplification of host type I interferon signaling with the direct inhibition of NP-associated vRNP trafficking, indicating that it functions as a coordinated antiviral regulator rather than as a single antiviral effector. From a translational perspective, this dual-layered regulatory mechanism may provide a conceptual framework for developing RNA-based antiviral strategies against avian influenza. Nevertheless, its physiological relevance and therapeutic potential require further validation in animal models.

In summary, this study identifies ckATP1A1-AS1 as a multifunctional antiviral regulator that restricts IAV replication via a dual strategy of potentiating innate immune signaling and directly interfering with the viral life cycle. These findings both expand our understanding of the functional diversity of avian antisense lncRNAs and provide a robust theoretical foundation for the development of novel antiviral therapeutic targets.

## MATERIALS AND METHODS

### Cells and viruses

Chicken embryo fibroblast (DF-1) cells, Madin Darby canine kidney (MDCK) cells, human lung adenocarcinoma (A549) cells, mouse lung epithelial-12 (MLE) cells, leghorn male hepatoma (LMH) cells, and human embryonic kidney (HEK293T) cells were cultured in Dulbecco’s modified Eagle’s medium (DMEM; Gibco, Thermo Fisher Scientific, Waltham, MA) containing 10% fetal bovine serum (FBS; SenBeiJia Biological Technology, Nanjing, China), 0.2% NaHCO3, 100 μg/mL streptomycin, and 100 IU/mL penicillin (Thermo Fisher Scientific, USA) at 37°C with 5% CO_2_. Primary chicken embryo fibroblast (CEF) cells were isolated from 9- to 10-day-old specific pathogen-free (SPF) chicken embryos, digested with trypsin, and maintained in DMEM medium supplemented with 5%–10% FBS at 37°C in 5% CO_2_.

Influenza viruses A/WSN/33 (H1N1) and A/Hong Kong/1/68 (H3N2) were grown on MDCK cells. A/chicken/Anhui/LH99/2017 (H9N2), A/duck/China/H6N6/2019 (H6N6), A/chicken/Jiangsu/0169/2021 (H10N3), A/Henan/4-10/2022 (H3N8), and Newcastle disease virus (NDV) LaSota strain (lentogenic, genotype II) strains were propagated in 10-day-old SPF embryonated chicken eggs. Viral titers were measured via plaque assays in MDCK cells.

### Antibodies, plasmids, and siRNA

Antibodies against NP, PB1, PB2, and PA were generated in our laboratory. The following antibodies were purchased: anti-FLAG (AE092) and anti-MYC (AE070) antibodies from Abclonal; anti-phospho-TBK1 (S172) antibody (T58364S) from Abmart; anti-phospho-IRF7 (Ser471/472) antibody (5184S) from CST; anti-IRF7 polyclonal antibody (22392-1-AP), anti-TBK1 polyclonal antibody (28397-1-AP), anti-OAS1 polyclonal antibody (14955-1-AP), anti-MX1 polyclonal antibody (13750-1-AP), anti-IFITM1 polyclonal antibody (11727-3-AP), anti-ISG15 polyclonal antibody (15981-1-AP), and anti-GAPDH antibody (60004-1-Ig) from Proteintech.

The ckATP1A1-AS1 sequence was inserted into the pCDNA3.1 vector. The open reading frames (ORFs) of H9N2-derived PB2, PB1, PA, and NP were cloned into the pCAGGS expression vector. The plasmids pCAGGS-FLAG-PB1, pCAGGS-FLAG-PB2, pCAGGS-FLAG-PA, and pCAGGS-FLAG-NP were generated via insertion of the H9N2-derived PB1, PB2, PA, and NP open reading frames. For the generation of pcDNA3.1-MYC-importin α1, pcDNA3.1-MYC-importin α3, pcDNA3.1-MYC-importin α5, and pcDNA3.1-MYC-importin α7, the importin sequence was fused to an MYC tag sequence at the N-terminus into the pcDNA3.1 vector. The plasmid constructs were confirmed by sequencing. The following siRNAs targeting ckATP1A1-AS1 (si-ckATP1A1-AS1, sense: 5′-GUCUAAAGUACUUCCAUAATT-3′; antisense: 5′- UUAUGGAAGUACUUUAGACTT-3′), targeting JUN (si-JUN, sense: 5′-CAGUCCAGCAACGGGUUAATT-3′; antisense: 5′-UUAACCCGUUGCUGGACUGTT-3′), targeting IRF7 (si-IRF7, sense: 5′-CUUCGAGCUUAUCGAGCAATT-3′; antisense: 5′- UUGCUCGAUAAGCUCGAAGTT-3′), and the antisense oligonucleotide (ASO) targeting ckATP1A1-AS1 (5′-TAACACACAGAAGCCCGTAA-3′) were purchased from GenePharma (Shanghai, China).

### Preparation of cell samples for RNA library construction

To prepare the viruses, six cell culture dishes (10 mm) were seeded with DF-1 cells. Once the cells reached 100% confluence, three dishes were designated as the experimental group, and the cells were infected with the H9N2 virus at an MOI of 3. The virus was allowed to attach for 1 h at 37°C; the viral supernatant was then discarded, and DMEM containing 0.35 µg/mL TPCK trypsin, 0.3% bovine serum albumin (BSA), and 25 nM HEPES was added to continue culture. The remaining three dishes were used as controls (without viral infection); in the controls, the cell culture medium was replaced with cell maintenance medium, and the remaining steps were performed identically.

To collect the adherent cells, the culture medium was discarded 12 h after infection, and 1 mL of Total RNA Extraction Reagent (R401, Vazyme, Nanjing, China) was added to the cells at 1 mL per 10 cm² of culture area. The cell lysate was transferred to RNase-free Eppendorf tubes using a pipette, and the cells were repeatedly pipetted using a 1 mL syringe until no clumps of cells remained, resulting in a clear and non-viscous solution. The samples were sent to Guangzhou Gene Denovo Biotechnology (Guangzhou, China) for transcriptomic sequencing.

### LncRNA library construction

A strand-specific library construction protocol was used. For enrichment of non-coding RNA transcripts, the Ribo-Zero Plus kit was used to remove ribosomal RNA from total RNA. The RNA was then randomly fragmented using a fragmentation buffer and used as a template to synthesize the first strand of cDNA using random primers; during the synthesis of the second strand, dUTP was substituted for dTTP to label the strand. Following end repair, A-tailing, and the addition of sequencing adapters, the cDNA strand containing dUTP was specifically degraded using the USER enzyme, thereby preserving transcript orientation. Finally, library construction was completed via PCR amplification and magnetic bead purification. After performing quality control using an Agilent 2100 Bioanalyzer instrument and qPCR, the library was sequenced using the Illumina platform.

### Analysis of lncRNA sequencing data

The raw sequencing data were first processed using Fastp or Trimmomatic to remove adapter sequences and low-quality reads, yielding high-quality, clean data. HISAT2 was used to perform oriented alignment of the clean data to the reference genome, and StringTie was employed to reconstruct transcripts from the alignment results. Subsequently, by comparing the data against known gene databases (such as Ensembl and GENCODE), new transcripts located in intergenic regions, introns, or antisense strands were identified. To identify potential lncRNAs, the protein-coding potential of candidate transcripts was predicted using the CPC2 and CNCI databases, and sequences with coding potential were excluded (the screening threshold was set to retain transcripts with length ≥200 bp and number of exons ≥2). Finally, quantitative analysis was performed using RSEM, and differentially expressed lncRNAs were identified using DESeq2, using the typical screening criteria log₂ (fold change) ≥1 and false discovery rate (FDR) ≤ 0.05.

### Bioinformatics analyses

Pearson’s correlation coefficients between two parallel replicates were calculated to evaluate the repeatability and reliability of the experiments. Principal component analysis was performed using R software (v4.0.0; R Foundation for Statistical Computing, Vienna, Austria) with the *prcomp* package to visualize sample similarity. A correlation coefficient closer to 1 indicates higher reproducibility between replicates. Gene Ontology (GO) functional classification and enrichment analysis of DEGs were carried out using the GO database (https://www.geneontology.org/). Significantly enriched GO terms, encompassing molecular function, cellular component, and biological process, were identified using the hypergeometric test against the genomic background. KEGG pathway enrichment analysis was performed using the KEGG database to identify significantly enriched metabolic or signal transduction pathways in DEGs. Significance was determined using the hypergeometric test with FDR ≤ 0.05, following the same statistical framework used for the GO analysis.

### Transient transfection

Cells were transiently transfected with siRNA or DNA plasmids in 24-well plates using Lipofectamine 2000 Reagent (Invitrogen, Thermo Fisher Scientific) according to the manufacturer’s protocol. For functional analysis, cells were transfected with either overexpression plasmids (500 ng per well) or control vectors (500 ng per well), alongside the siRNA (50 nM, GenePharma) in culture medium.

### Plaque assay

IAV infectious titers were determined via a plaque assay. Briefly, the viral supernatants were serially 10-fold diluted with DMEM and inoculated onto an MDCK cell monolayer. After incubation at 37°C for 1 h, the cells were overlaid with 2× DMEM containing 2% plaque agarose (Lonza, Basel, Switzerland) and 1 μg/mL TPCK trypsin, and then placed upside down at 37°C. At 36–48 hpi, the quantity of plaques was detected.

### Western blotting

The cells were washed with phosphate-buffered saline (PBS) and lysed in NP-40 lysis buffer, after which protease and phosphatase inhibitors were added; the cells were then kept on ice for 30 min, with intermittent vortex mixing. The lysates were centrifuged at 12,000 × *g* for 15 min at 4°C. The supernatants were collected, and protein concentrations were quantified via a bicinchoninic acid assay. Equal amounts of protein (20–40 μg) were mixed with 5× SDS sample buffer, denatured at 95°C for 5 min, and separated by SDS-PAGE on 10%–12% resolving gels. Proteins were separated via electrophoresis and transferred to 0.22 μm nitrocellulose membranes via wet transfer at 80 V for 90–120 min. The membranes were blocked using 5% non-fat milk or BSA in TBST for 1 h at room temperature, then incubated with the primary antibodies (1:1,000) overnight at 4°C. After three 10-min TBST washes, the membranes were incubated with horseradish peroxidase-conjugated secondary antibodies for 1 h at room temperature. Following additional washes, the protein bands were visualized using an enhanced chemiluminescence substrate and detected using a chemiluminescence imaging system. GAPDH or β-Actin served as loading controls for normalization. Quantitative analysis of western blot bands was performed using the ImageJ software (National Institutes of Health, Bethesda, MD, USA).

### RNA isolation and RT-qPCR

Total RNA was extracted from cells using Total RNA Extraction Reagent (R401, Vazyme) and validated using a NanoDrop spectrophotometer. RT-qPCR was performed using the 2× SYBR Green Pro Taq HS Premix (AG11701, Accurate Biology). Reactions were carried out in a Roche Light Cycler 96 (Roche, Basel, Switzerland), according to the manufacturer’s instructions, using the following cycling program: 95°C for 5 min, 40 cycles at 95°C for 10 s, and 60°C for 30 s. GAPDH or U6 was used as the endogenous reference gene, and the relative RNA levels were calculated using the 2^−ΔΔCt^ method. The primer sequences are provided in Tables S1 and S2.

### IAV attachment and internalization assay

DF-1 cells were seeded into 6-well plates and transfected with pcDNA3.1-ckATP1A1-AS1 or the empty vector. After 24 h, the cells were infected with H9N2 (MOI = 5) and incubated at 4°C for 1 h. For the virus-attachment assay, the cells were washed three times with cold PBS (pH, 7.4) to remove unbound virus.

For the virus internalization assay, the infected cells were incubated at 4°C for 1 h to allow for viral binding, then the infected cells were washed three times with cold PBS (pH, 7.4) to remove any unbound virus. Subsequently, the cells were incubated in prewarmed Opti-MEM for 1 h at 37°C to allow for viral endocytosis and then washed three times with cold PBS (pH = 1.5) to remove non-internalized virions.

In both assays, the cell lysates were harvested, and the vRNA levels of viral NP and protein levels of viral PB1 and NP were determined using RT-qPCR and western blotting respectively.

### Luciferase reporter assay

HEK293T cells were co-transfected for 36 h with PCAGGS-PB2, PCAGGS-PB1, PCAGGS-PA, PCAGGS-NP, pHH21-PolI-Luc, and pcDNA3.1-ckATP1A1-AS1, using pRL-TK (*Renilla*) as the internal control. Cell lysates were collected to measure the firefly and *Renilla* luciferase activities. Relative luciferase activity was normalized to that of *Renilla*.

### Viral polymerase-minigenome assay

To evaluate the influence of ckATP1A1-AS1 on influenza polymerase activity, HEK293T cells in 24-well plates were transfected with 100 ng of pCAGGS plasmids encoding the proteins PB2, PB1, PA, and NP of AH99, together with 50 ng of a species-specific minigenome reporter plasmid (pHH21-PolI-vLuc), 50 ng *Renilla* luciferase expression plasmid (pRL-TK) as the internal control, and a pcDNA3.1-ckATP1A1-AS1 (250 ng), using Lipofectamine 2000 Reagent (Invitrogen) according to the manufacturer’s instructions. After incubation at 37°C for 48 h, the cells were lysed with 100 μL of the passive lysis buffer (Vazyme), and firefly and *Renilla* luciferase bioluminescence were measured using a Dual-Luciferase system (Vazyme).

### Immunofluorescence assays

DF-1 cells grown in culture dishes were transfected with plasmids or siRNA, or these cells were infected with the H9N2 virus, as indicated. The cells were fixed with 4% paraformaldehyde for 30 min, permeabilized with 0.2% Triton X-100 for 10 min, and blocked with 2% BSA for 1 h at room temperature. Incubation with the corresponding primary antibodies was carried out at 4°C overnight, followed by further incubation with secondary antibodies conjugated to Alexa Fluor 488 or Alexa Fluor 561 for 1 h at room temperature. After three washes, the cells were incubated with DAPI (4′,6-diamidino-2-phenylindole; Thermo Fisher Scientific, Waltham, MA, USA) for 10 min at room temperature to stain the nuclei. Images were visualized using a confocal microscope (Nikon, Japan), and protein fluorescence intensities were quantified using ImageJ software.

### Co-immunoprecipitation assay

HEK293T cells were grown to approximately 70%–80% confluence in 6-well plates, and then transfected with the indicated plasmids, using the Lipofectamine 2000 Reagent (Invitrogen). After 48 h, the cells were lysed on ice for 30 min with IP lysis buffer (Pierce, Rockford, IL, USA) containing a protease inhibitor cocktail on ice for 30 min, and then centrifuged at 12,000 × *g* at 4°C for 10 min. The supernatants were incubated with the corresponding primary antibodies at 4°C overnight, followed by the addition of protein A/G beads and incubation at 4°C with rotation. After 6 h, the beads were washed three times with cold PBS. The immunoprecipitated proteins were then separated by SDS-PAGE and transferred onto nitrocellulose for Western blotting.

### RNA immunoprecipitation assay

DF-1 cells were grown to approximately 70%–80% confluence in 6-well plates and then transfected with the indicated plasmids and lysed in IP lysis buffer. Protein A/G beads were incubated with IgG and anti-FLAG antibody at room temperature for 2 h and mixed with the cell lysates via rotation at 4°C overnight. After the protein A/G beads had been washed with PBST (0.02% Tween 20 in PBS) five times, the co-precipitated RNA was extracted with Trizol LS reagent, and ckATP1A1-AS1 and GAPDH levels were detected by RT-qPCR.

### RNA protein pulldown assay

For the RNA pull-down assay, DF-1 cells were grown to approximately 70%–80% confluence in 6-well plates and then transfected with the pcDNA3.1-ckATP1A1-AS1 plasmid. Biotin-labeled sense or antisense probes (GenePharma) were incubated with the cell lysates, followed by overnight binding with streptavidin magnetic beads at 4°C. The Pierce Magnetic RNA-Protein Pull-Down kit (Thermo Scientific) was used for RNA pull-downs, according to the manufacturer’s instructions. The protein eluates were separated by 10% SDS-PAGE and stained with a silver stain kit (Beyotime Technology, Shanghai, China).

### Chromatin immunoprecipitation assay

Chromatin immunoprecipitation (ChIP) was performed using the BeyoChIP Kit (Beyotime, P2080S), following the manufacturer’s protocol. Briefly, the cells were cross-linked with 1% formaldehyde for 10 min at 37°C and quenched with 0.125 M glycine. The harvested cells were lysed in SDS lysis buffer containing protease inhibitors. The chromatin was sheared into 200–1,000 bp fragments via sonication on ice. After centrifugation, the supernatant was diluted and pre-cleared with protein A/G magnetic beads (blocked with salmon sperm DNA). The chromatin was then incubated with anti-FLAG antibody or normal IgG (as the negative control) overnight at 4°C. Immune complexes were captured with magnetic beads and washed sequentially with low salt, high salt, LiCl, and TE buffers. DNA was eluted with elution buffer (1% SDS, 0.1 M NaHCO₃), and cross-linking was reversed at 65°C for 4 h. Following proteinase K digestion, the ChIP DNA was purified via phenol-chloroform extraction and analyzed using RT-qPCR.

### Fluorescence *in situ* hybridization assay

RNA fluorescence *in situ* hybridization (FISH) assay was performed using the RNA FISH Kit (SA-Biotin System, GenePharma). Briefly, cells cultured on coverslips were fixed with 4% paraformaldehyde for 15 min at room temperature, followed by permeabilization with 0.1% Triton X-100 for 15 min. After blocking and equilibration with 2× saline sodium citrate (SSC), the samples were incubated with a hybridization mixture containing 1 μM biotinylated probes pre-conjugated with streptavidin-Cy3 (SA-Cy3) at 37°C overnight in the dark. After hybridization, the coverslips were washed sequentially with 0.1% Tween-20 at 37°C and 2× SSC at 60°C to remove nonspecific binding. Nuclei were counterstained with DAPI for 10 min. Finally, the slides were mounted with anti-fade mounting medium and visualized using a fluorescence microscope. The following biotinylated probe to ckATP1A1-AS1 was used: 5′-CTTATCGTGCGTGCCCTGTTA-3′ (synthesized by Genepharma).

### Cell fractionation

The nuclear and cytoplasmic fractions were isolated using the PARIS Kit (Invitrogen), according to the manufacturer’s instructions. Briefly, the cells were harvested, washed with ice-cold PBS, and resuspended in Cell Fractionation Buffer. After incubation on ice for 5–10 min, the lysate was centrifuged at 500 × *g* and 4°C for 5 min to pellet the nuclei. The supernatant, containing the cytoplasmic fraction, was carefully transferred to a fresh tube. The nuclear pellet was washed gently with Cell Fractionation Buffer and subsequently lysed in Cell Disruption Buffer to obtain the nuclear fraction. For RNA or protein analysis, the respective fractions were immediately processed with the provided lysis/binding buffers or stored at −80°C until further use. Fraction purity was validated by detecting specific markers (GAPDH for cytoplasm and U6 for nucleus) via western blotting or RT-qPCR.

### Animals

Specific-pathogen-free embryonated chicken eggs were obtained from Jinan Sais Poultry Technology Co., Ltd. (Jinan, China). Primary CEF cells were isolated from 9- to 11-day-old chicken embryos.

### Statistical analysis

The data are shown as the mean ± standard deviation (SD), and unless otherwise indicated, all presented data are representative results of at least three independent repeats. Statistical analysis was performed with Prism 9 (GraphPad), using two-tailed Student’s *t-*tests or one-way or two-way ANOVA as indicated. Differences considered to be significant are indicated by asterisks as follows: *, *P* < 0.05; **, *P* < 0.01; ***, *P* < 0.001; ****, *P* < 0.0001. “ns” indicates no significance.

## Data Availability

The high-throughput lncRNA sequencing data set analyzed in this study can be accessed through the NCBI SRA database under the accession number PRJNA1466733. All other raw experimental data are available from the corresponding author upon reasonable request.

## SUPPLEMENTAL MATERIAL

Supplemental material jvi.00259-26-s0001.docx Fig. S1 to S8, Tables S1 and S2, sequence information, and RNA sample inspection report.

Table S3 jvi.00259-26-s0002.xlsx Sequencing quality-control metrics of lncRNA-seq libraries.

Table S4 jvi.00259-26-s0003.xlsx rRNA removal and reference genome mapping statistics of lncRNA-seq libraries.

Table S5 jvi.00259-26-s0004.xlsx High-throughput lncRNA sequencing data.

ASM does not own the copyrights to Supplemental Material that may be linked to, or accessed through, an article. The authors have granted ASM a non-exclusive, world-wide license to publish the Supplemental Material files. Please contact the corresponding author directly for reuse.
