# Supplementary material for: LncRNA ckATP1A1-AS1 inhibits influenza A virus replication by mediating innate immune responses and suppressing viral nuclear import
Source: J Virol. 2026 Jul 24;100(8):e00259-26. doi: 10.1128/jvi.00259-26 (PMC13483405; doi:10.1128/jvi.00259-26)
Supplement: Supplementary file 1 [file jvi.00259-26-s0001.docx]

**Supplementary Contents**

**1. Supplementary Figure S1-S8**

**2. Supplementary Table S1-S2**

**3. LncRNA gene sequence information**

**4. RNA sample inspection report**

**1. Supplementary Figures**

**Supplementary Fig. S1.** Predicted secondary structure of ckATP1A1-AS1 generated using RNAFold.

**Supplementary Fig. S2.** ckATP1A1-AS1 inhibits IAV replication in DF-1 cells. (A) Knockdown efficiency of ckATP1A1-AS1 in DF-1 cells transfected with specific siRNAs or negative control (si-NC), as determined by RT-PCR. (B) DF-1 cells with ckATP1A1-AS1 silenced were infected with H9N2 virus at an MOI of 0.1. Cell supernatants were collected at 12, 24, and 36 hours post-infection, and viral titers were determined by plaque assay. (C) Knockdown efficiency of ckATP1A1-AS1 in DF-1 cells transfected with specific ASO or negative control (NC), as determined by RT-PCR. (B) DF-1 cells with ckATP1A1-AS1 ASO-knockdown were infected with H9N2 virus at an MOI of 0.1. Cell supernatants were collected at 12, 24, and 36 hours post-infection, and viral titers were determined by plaque assay. (E) The overexpression efficiency of ckATP1A1-AS1 in DF-1 cells transfected with pcDNA3.1-ckATP1A1-AS1 or the empty vector was determined by RT-PCR. (F) DF-1 cells with ckATP1A1-AS1 overexpressed were infected with H9N2 virus at an MOI of 0.1. Cell supernatants were collected at 12, 24, and 36 hours post-infection, and viral titers were determined by plaque assay. (G) Rescue assay confirmed that ckATPA1-AS1 inhibits IAV replication. After knockdown the ckATPA1-AS1gene (si-ckATPA1-AS1) and overexpressing ckATP1A1-AS1 in DF-1 cells, respectively, or applying both treatments simultaneously, the cells were infected with H9N2 virus (MOI = 0.1) for 24 hours and viral titers were determined by plaque assay. Statistical differences between groups were marked according to one-way analysis of variance. Data are presented as the mean ± standard deviation (SD) of three independent experiments. *, *P* < 0.05; **, *P* < 0.01; ***, *P* < 0.001; ****, *P* < 0.0001; “ns” indicates no difference.

**Supplementary Fig. S3.** Biological Characteristics of ATP1A1. (A and B) Expression kinetics of the host gene ATP1A1 during IAV infection. ATP1A1 mRNA levels were measured by RT-qPCR at indicated time points (A), and ATP1A1 protein levels were analyzed by western blot (B). (C) DF-1 cells overexpressing ATP1A1 were infected with H9N2 virus at an MOI of 0.1 for 12, 24, and 36h, and viral titers were determined by plaque assay. Statistical differences between groups were marked according to one-way or two-way analysis of variance. Data are presented as the mean ± standard deviation (SD) of three independent experiments. *, *P* < 0.05; **, *P* < 0.01; ***, *P* < 0.001; ****, *P* < 0.0001; “ns” indicates no difference.

**Supplementary Fig. S4.** Identification of mRNA and protein expression of JUN gene. (A-B) mRNA levels of JUN in DF-1 cells infected with IAV at indicated time points (A) and doses (B). (C) Western blot analysis of total JUN and phosphorylated JUN (p-JUN) levels in cells infected with IAV at 0, 6, 12, 24, 36 and 48hpi. Results were normalized to GAPDH. Statistical differences between groups were marked according to one-way analysis of variance. Data are presented as the mean ± standard deviation (SD) of three independent experiments. *, *P* < 0.05; **, *P* < 0.01; ***, *P* < 0.001; ****, *P* < 0.0001; “ns” indicates no difference.

**Supplementary Fig. S5.** The rescue experiment confirmed the regulatory relationship between IRF7 and ckATPA1-AS1. (A and B) After knockdown the IRF7 gene (si-JUN) and overexpressing ckATP1A1-AS1 in DF-1 cells, respectively, or applying both treatments simultaneously, the cells were infected with H9N2 virus (MOI=0.1) for 24 hours. (A) RT-qPCR was used to detect the expression levels of IFN-β mRNA. (B)Viral titers were determined using plaque assay. All shown RT-qPCRs results were normalized to GAPDH. Statistical differences between groups were marked according to one-way analysis of variance. Data are presented as the mean ± standard deviation (SD) of three independent experiments. *, *P* < 0.05; **, *P* < 0.01; ***, *P* < 0.001; ****, *P* < 0.0001; “ns” indicates no difference.

**Supplementary Fig. S6.** HuATP1A1-AS1 exhibits no regulatory effect on the replication of influenza virus. (A) Predicted secondary structure of huATP1A1-AS1 generated using RNAfold. (B) Homologous Secondary Structure Prediction of ckATP1A1-AS1 and huATP1A1-AS1 Using RNAalifold. (C) A549 cells with huATP1A1-AS1 overexpression were infected with H1N1 virus at an MOI of 0.1. Cell supernatants were collected at 12, 24 and 36 hours post-infection, and viral titers were determined by plaque assay. Statistical differences between groups were marked according to two-way analysis of variance. Data are presented as the mean ± standard deviation (SD) of three independent experiments. *, *P* < 0.05; **, *P* < 0.01; ***, *P* < 0.001; ****, *P* < 0.0001; “ns” indicates no difference.

**Supplementary Fig. S7.** KEGG pathway enrichment analyses. Sankey diagrams showing that trans genes participate in lysosome, apoptosis, protein processing endoplasmic reticulum and FoxO signaling pathways.

**Supplementary Fig. S8.** mRNA levels of potential genes related to ck-ATP1A1-AS1 expression. (A) Effects of ckATP1A1-AS1 overexpression on the transcription of potential genes, as analyzed by RT-qPCR. (B) Effects of ckATP1A1-AS1 knockdown on the transcription of potential genes, as analyzed by RT-qPCR. Results were normalized to GAPDH. Statistical differences between groups were marked according to two-way analysis of variance. Data are presented as the mean ± standard deviation (SD) of three independent experiments. *, *P* < 0.05; **, *P* < 0.01; ***, *P* < 0.001; ****, *P* < 0.0001; “ns” indicates no difference.

**2. Supplementary Tables**

**Table S1.** The primer sets for RT-qPCR

| **Gene (chicken)** | **Primers** |
| --- | --- |
| Gallus-GAPDH | Forward: 5’- GAGGGTAGTGAAGGCTGCTG -3’  Reverse: 5’- CACAACACGGTTGCTGTATC-3’ |
| Gallus-U6 | Forward: 5’- GATAATACTGGGGACAAGCAG-3’  Reverse: 5’- CACCCAAATTCGTATGTCATCC-3’ |
| ckATP1A1-AS1 | Forward: 5’- TGGAGGATCACAGGATGACAGA-3’  Reverse: 5’- TTCAAGGAGCGTTTGGACAG-3’ |
| Gallus-IFN-β | Forward: 5’-CCAGCTCCTTCAGAATACGG-3’  Reverse: 5’-TGGCTGCTTGCTTCTTGTCC-3’ |
| Gallus-Mx1 | Forward: 5’- GCTGTTGCGATGCTGAACAA-3’  Reverse: 5’- AACCAATTCTGGCCTGAGCA-3’ |
| Gallus-IFIT5 | Forward: 5’- CTAGGACTCTGCTACCGAGC-3’  Reverse: 5’- TTGCCTCCGCATACATCCTT-3’ |
| H9N2-NP-vRNA | Forward: 5’-GGCCGTCATGGTGGCGAAT-3’  Reverse: 5’-GGCTGTTTTGAAGCAGACGG-3’ |
| H9N2-NP-cRNA | Forward: 5’-GGCAACGAACCCGATCGTG-3’  Reverse: 5’-GCTAGCTTCAGCTAGGCATC-3’ |
| H9N2-NP-mRNA | Forward: 5’-GGCAACGAACCCGATCGTG-3’  Reverse: 5’-CCAGATCGTTCGAGTCGT-3’ |
| Gallus-ATP1A1 | Forward: 5’-AGGGCAACGAAACAGTGGAA-3’  Reverse: 5’-ACATGCCTTAGCATCCCTCG-3’ |
| Gallus-JUN | Forward: 5’-AGCAACGGGTTAATCACCAC-3’  Reverse: 5’-GCAGCGTGTTCTGGTTGT-3’ |

**Table S2.** Primers for RNA reverse transcription

| **Gene** | **Primers** |
| --- | --- |
| NP-vRNA-RT | GGCCGTCATGGTGGCGAATGCAAGGTCTGCACTCATCCT |
| NP-cRNA-RT | GCTAGCTTCAGCTAGGCATCAGTAGAAACAAGGGTATTTTTCTTT |
| NP-mRNA-RT | CCAGATCGTTCGAGTCGTTTTTTTTTTTTTTTTTCTTTAATTGTC |

**3. LncRNA gene sequence information**

**ckATP1A1-AS1**

CTGGCAGGCCCGATAGCTCAGCTGGTAAACGTGCAGGGCTGATAACACCAGGTCACACGCTCGAGTCCCATGTGGGCTCCTCTCTTTCCACTCCAACAGCTCGAACCAATGACCCTTATGGCACTCCGTGATTACATCCAACCTCGAGCTCCTGCTTGAGAGCCTCCAAACATTTAAGAGAGAACCTGGACGGAGGACTTCTTACTCCGGGCTGTAACATGGAGAACGGAGGGAAGGCAGGCTGACACTGCCCTTGCACACACACACACACACACACACCACTGCTGCACAGCATGCCGGGAGGCTATCATCTCAATTAACAGGGCACGCACGATAAGGTCACGCCTGATCGCCTTCCTTATGCCATCAGCTCCTATCTGCCCTCAACGCCGCTAACGCGCAGCGCCACGCGCTGCCAACTCCTTCCCCTCCGGGGCCCGGCGGCCGCAAGGAGTCTAAAGTACTTCCATAAGGGCCAGGAGACGTCGGGGCAGCCGCACTTCCCAAAAGGGCACCAGATAACACCGCCCGCGGGGAACACGCCCGTTCCCACACCGCCCTCACCTGCAGCCCCGCGGTGCCGGTCGGCTTCACGCCTCACCGCGCGGCGAGGGTCCCCCCCCCCCCCCCGCGGCCATTTCCAGCGCCGCTCGGGCTCCCCCACCCTCCGGCAACGCCCGCGGGGAGCCGCCTCCCGGCGCCGGCCCCGGGCCGGCGCTGCCCCCTCGTTACCTCCTGGGGCCGCCGCTTCCTCCCCCGAGCCCGGCACTGAGCCGGCCGGAGCCGCCCCGCCGCCCGGCACCCCGCACGATCCCCGCACCCGGCCCGGCCGCTCACCCCCTTCCCCATGGTGCCGGGCGTGCGGGACACGCGGCTGGCAGCGAGGAGCGGCGCTCAACGACGCCACGTGGCTCAGCAGGTGCCGAACAATAGCAGCGCCCGCCGCTCCGCTTTCCTCCCGCTCGCCTCTCTCTCGCCCGGCTCCTCGCCGCCGCAACCACCCGCAGCTCCGATACGGCACCGGCTTCCCGCCGGATAAAGCCCCGGCCGCCCCGCCCCGTCCCGCCCGTAGCCCGCCCCTCCAAGCGGACCGTTGGGGCGGGGGGGGTGGTGCCGGCTTGCCCACGTGTAAAGCGGGGCTCACCTCGGCCGCCCCAACCGCGCCCCACCGAGGAGGGCTCGGCGCGTCGCCGCGGGCTCCTTCCGGGCCCGGGGTGAGGCCGCCTGAGGGCGCTGCGCTGCCCCGTTCCCGCCTTGCTGGCGCTGCCGCCGCCGCCATGTCGAGGCGGTGAGGGGGCAGTGGGCGCGGCGCCCCCGCTGCTCTGCCGCGCTCCGGTTCGTGGCACTGCGTTCGCTGAAAGGCACCGGGGTGAAGCCAGGCGCGGCCACAGGACCCTGCTCTGAGGGCACCACCAGGGGTCCCTTCCTGCCTACAGCCACACAGCCTGCTGGAGTGACGTGGTGTCCGAAAGGCTGCGCACCTTCCGGGGTTACGGGCTTCTGTGTGTTAGGAGATGCGTATGCGCTCGTCAAAACGGCGTTGTGCTCTCTGTGCTCTGCTGCTCAGTGCCTGGCAGGGCAATGGTCTCATAGGTCCCTTCCCACTCGAGCACATGGGAGAGATGGGCAGATGGAGGATCACAGGATGACAGAAACACAGAATATCCCGAGTTGGAAGGGACACATAAGGATCATCGAGTGCAACCCCTGGCTCCACACAGCACTACCCAAAATCCAGACGCTATCTCTGAGAGCACTGTCCAAACGCTCCTTGAACTCCGGCAGCCTGGGGCCGTGCCCACTGCCCTGTGGTGAGGAACCTTCTCCTAACCCCCAAGCTGACCCCTCCCTGAAGCTGTAGCGCCATCGGTTTGTCCACAGAGCTGTGTGATGGGAAGGGATGCTGCTACAAATGGCAGTCTTAAAGGCTTTGGGGGGAGCATCACACACCGGTGGAGCTCAGTCTGGTCATTGTCCCCAAGTCCTCCTGAGGCACTCCTAGTACAGGACGTAGTGAACTTTAAAGTAAAAACCTGCACCATCCCGTCCCCAAGACAGGGCTGTCATTTCGCTCCCGTTACTCATCTCTGAAGTGTTCTTTCCCTTGAAAGT

**ENST00000369492 (huATP1A1-AS1)**

CGGAAGCTCCCACCTGAGCGGCGCATCCTCAGGGCCTGGGCCTGGCCTCCTTGCCTGTGAGATGGCACTGTGACGGTGCCCACCTCGTGGGGTGTGTGAGGGCCGAGTGAAATCGTGCATTTGCGATGTCTAGGACACACTGGATTTGTACATCTGGGGCTCAGGAGAGGACCAGATCAGAGCTGGAAATCATGCCAGGAAGAGTATGAAGCAGGGAGAGAAGGTGTGGAGGCCTCCCTGAGGAAACGAGGGGTCGAGCAGGTGAAGAGGGGGCTGAGAGCTAAGGAGTGCACAGAGACAATGACAGAAAGACGGGAGGTCTGGTTTCAGGAAGGGTGTGATGGACAGAAGAGATGCAGCAGAGGTCAAGGAAGGACCAAGATGCATCAGGAGGAAATGCCCATGACCTTCATCGCAGCAGCGTATGTGCGTTGGTCTTCTCCAGGGATGCCGAAAGTAGACATACAGGGAGTCGCAGTACGGATACCACCAGTGCCAGCCTGACCTCACAAGAGACGTCTTGTGTGACTGGCCCTGCCGTCACAGCTGGGCCAGTAAAGTACCTTCCAGGAGGCTCCAGTTGCTACCTGACTCACAACCTTCTAATCCCATGATCTTTTAGTTCTAGTTTCCTTATCTTGGTCTGAGCAAATGGCTCATTTTAAAGATGACTTGCAGACAAATGTAGAGATCATCCCAGGGGAAAGTGCCCCAAGAAAGGAGAGTCCAAGACCACCTGCCCCTCCCAGTTCTGCGGCTGGAGTGGGTGGGTGCAGCAACCACAGCCCCAGTGTACAGGAGTCACCACTGTCTCCTCCCGCATTGGCCCAGCTTGGAAGTGCACAGCAACCAAGCATGAGGACTGAGTTAAGCTTTTCGGAGAAAAAAGACACTATGATCATCTGGCAGATCACGATCACTGTTTGGTGCCAGAGGTAAGGAGTTTGCTTGTTGTGTTTGCTTGGAGGCGACAGTTGGCAATGACAGCCTGGGAGAAGCACCGGCAGTTAACAGCACTCTGCACCACTCATGGCTGAGACTGAGGGGAGCCTTTTACAGCAGCTCTGAAAGCAGGAGGTGTTCTCAGACTTGACCCTGAAGTAAACACATCTCTTTCACCTTTTTCTTAAGTGCTTGCTGGCACATAACCACTTGCTGAAATTGTGTCATTGATGCTAAATTAGGAACACATTATATGGAAGGAAGGGAACCACTTCTCAGCACACATGCCCAGGCAGACTGTGTGGGATAGATTTCTGCTTCTCAGATAAGCCCTGGGCTAGAGACACTTGAGATGTGGTGGTGATCTCTGGAGGGAAAGCAGGAAGGCTTCAGGTCTCTTTTAGACTGAAAGAGAAGGGCCTCATCTGCAGTTTAGTATACTCTGAGGGTCTGGAAATCCTTTAGGCGAGGTGCTCTTCCATCAAAAAGCCAGATGCTAAGGCAGCAGGTGGAGTTACAGCCCTATCACCCCAGGCAGCAGCCAGAAAAAATGGGTCAGGTAAACTCACCTACTCCAACTAAAAGAGCACGGGCAGAATGCTTCCTCTTAGCCAGTGGTTTCCAAACTTGAATGTAAATCAAAATCACCTGAGGGCTTATTAAGCCACATTGCTGATGCATTTCTGACTCAGTGGGTCTGGGCCCGCCTTCCTAGCAAGCAACCAGGTGATGCTGATAAACTGCTGGTCAGGGACTCATGTTGAAAACCACAGCTCCAGATCACATAGACATCTGTACCAAAGAAGCGACGAGGGCACAGACAAATTCCATTATAATAGCCATCTTTATTTGTAAAAATCCAGATATAAAATGTATTCTTTCAGTC

**RNA sample inspection report**

Sample concentration and RIN values are determined based on the results from Agilent 2100

Sample volume: 1 μL of stock solution or diluted solution

| **No.** | **Sample** | **Conc. ng/ul** | **Vol. ul** | **Tot. Amt. ug** | **RIN** | **Species** | **Type** |
| --- | --- | --- | --- | --- | --- | --- | --- |
| R22041630 | Mock-1 | 357 | 50 | 17.83 | 9.8 | Gallus | lncRNA |
| R22041631 | Mock-2 | 292 | 47 | 13.74 | 9.8 | Gallus | lncRNA |
| R22041632 | Mock-3 | 332 | 47 | 15.59 | 9.8 | Gallus | lncRNA |
| R22041633 | H9N2-1 | 248 | 46 | 11.42 | 9.8 | Gallus | lncRNA |
| R22041634 | H9N2-2 | 317 | 46 | 14.60 | 9.5 | Gallus | lncRNA |
| R22041635 | H9N2-3 | 311 | 46 | 14.30 | 9.6 | Gallus | lncRNA |
